# Self-Organization of Microcircuits in Networks of Spiking Neurons with Plastic Synapses

**DOI:** 10.1371/journal.pcbi.1004458

**Published:** 2015-08-20

**Authors:** Gabriel Koch Ocker, Ashok Litwin-Kumar, Brent Doiron

**Affiliations:** 1 Department of Neuroscience, University of Pittsburgh, Pittsburgh, Pennsylvania, United States of America; 2 Center for the Neural Basis of Cognition, University of Pittsburgh and Carnegie Melon University, Pittsburgh, Pennsylvania, United States of America; 3 Department of Mathematics, University of Pittsburgh, Pittsburgh, Pennsylvania, United States of America; 4 Center for Theoretical Neuroscience, Columbia University, New York, New York, United States of America; University College London, UNITED KINGDOM

## Abstract

The synaptic connectivity of cortical networks features an overrepresentation of certain wiring motifs compared to simple random-network models. This structure is shaped, in part, by synaptic plasticity that promotes or suppresses connections between neurons depending on their joint spiking activity. Frequently, theoretical studies focus on how feedforward inputs drive plasticity to create this network structure. We study the complementary scenario of self-organized structure in a recurrent network, with spike timing-dependent plasticity driven by spontaneous dynamics. We develop a self-consistent theory for the evolution of network structure by combining fast spiking covariance with a slow evolution of synaptic weights. Through a finite-size expansion of network dynamics we obtain a low-dimensional set of nonlinear differential equations for the evolution of two-synapse connectivity motifs. With this theory in hand, we explore how the form of the plasticity rule drives the evolution of microcircuits in cortical networks. When potentiation and depression are in approximate balance, synaptic dynamics depend on weighted divergent, convergent, and chain motifs. For additive, Hebbian STDP these motif interactions create instabilities in synaptic dynamics that either promote or suppress the initial network structure. Our work provides a consistent theoretical framework for studying how spiking activity in recurrent networks interacts with synaptic plasticity to determine network structure.

## Author Summary

The connectivity of mammalian brains exhibits structure at a wide variety of spatial scales, from the broad (which brain areas connect to which) to the extremely fine (where synapses form on the morphology of individual neurons). Recent experimental work in the neocortex has highlighted structure at the level of microcircuits: different patterns of connectivity between small groups of neurons are either more or less abundant than would be expected by chance. A central question in systems neuroscience is how this structure emerges. Attempts to answer this question are confounded by the mutual interaction of network structure and spiking activity. Synaptic connections influence spiking statistics, while individual synapses are highly plastic and become stronger or weaker depending on the activity of the pre- and postsynaptic neurons. We present a self-consistent theory for how activity-dependent synaptic plasticity leads to the emergence of neuronal microcircuits. We use this theory to show how the form of the plasticity rule can govern the promotion or suppression of different connectivity patterns. Our work provides a foundation for understanding how cortical circuits, and not just individual synapses, are malleable in response to inputs both external and internal to a network.

## Introduction

The wiring of neuronal networks exhibits structure across a broad range of spatial scales [1]. For example, patterns of connectivity among small groups of cortical neurons are over- or under-represented compared to random networks [2–5]. The prevalence of these motifs is related to neurons’ stimulus preferences and activity levels [6, 7]. Motivated in part by these observations, there is a growing body of theoretical work that discusses how wiring structure dictates the coordinated spiking activity of cortical neurons in recurrent networks [8–18].

While neural architecture undoubtedly plays a strong role in determining neuronal activity, the reverse is also true. Individual synapses can both potentiate (strengthen) and depress (weaken), and whether they do so depends on the relative timing of action potentials in the connected neurons [19, 20]. Such *spike timing-dependent plasticity* (STDP) has featured prominently in both experimental and theoretical studies of neural circuits [21–23]. Of particular interest, STDP provides a mechanism for Hebbian plasticity: synaptic potentiation occurs when a presynaptic neuron reliably drives spike responses from a postsynaptic neuron, while anti-causal spike pairs result in synaptic depression [24]. Hebbian plasticity provides a potential link between circuit structure and function through the formation of heavily wired assemblies of neurons, where assembly membership is associated with coordinated, elevated firing rates during a specific computation [25]. Evidence supporting this idea, originally proposed by Hebb [26], has been found in both hippocampus [27] and sensory cortex [28].

Despite the promise of STDP to provide insight into the functional wiring of large neural circuits, many studies of STDP have concentrated on the plasticity of synaptic connections between just a single pair of pre- and postsynaptic neurons, often focusing on the distribution of individual synaptic weights [24, 29–32]. Other studies have shown that multiple temporally correlated inputs to a neuron will cooperate to potentiate, while uncorrelated inputs may depress [24, 33–35]. In this case STDP can generate feedforward circuits [36], which while important for the propagation of neural activity [37], are unlike the recurrent structure of the neocortex. Understanding the two-way interaction between plastic recurrent network structure and spiking activity recruited in recurrent circuits is thus a central focus for theories of synaptic plasticity.

Due to this challenge, many studies have resorted to large-scale numerical simulations of cortical networks with plastic synapses [38–41]. While intuition for the development of circuit structure can be gained using this approach, without a governing theoretical framework it is often difficult to extract generalized principles. Alternatively, mathematical analyses have been restricted to either small networks [40, 42], or have required the assumption that neurons fire as Poisson processes [43–46]. These latter works assumed shared inputs from outside the network to be the only source of correlated spiking activity, neglecting covariance originating from recurrent coupling. Thus, there is a need for a coherent mathematical framework that captures how STDP drives self-organization of circuit structure in recurrent cortical networks.

To this end, we construct a self-consistent theory for the coevolution of spiking statistics and synaptic weights in networks with STDP. This theory makes use of a previously developed linear response framework for calculating joint spiking statistics [15, 47, 48] and a separation of timescales between spiking covariance and synaptic plasticity [33]. We then use this high-dimensional theory to derive a low-dimensional, closed system for STDP of two-synapse connectivity motifs in recurrent networks. This reveals instabilities in the motif dynamics such that when potentiation and depression are approximately balanced, the dynamics are partitioned into regimes in which different motifs are promoted or suppressed depending on the initial network structure. It also highlights the circumstances in which spike time covariations, in contrast to firing rates, drive STDP. In total, we provide a consistent and general framework in which to study STDP in large recurrent networks.

## Results

Our study is separated into two main sections. The first presents a self-consistent theory for spike timing-dependent plasticity (STDP) in recurrent networks of model spiking neurons. The second part leverages our theory to develop a low-dimensional dynamical system for the development of two-synapse motifs in the network structure. We analyze this system and determine how the balance between synaptic potentiation and depression drives the emergence of microcircuits in recurrent networks.

### Spike train covariance determines synaptic plasticity

**Fig 1:**
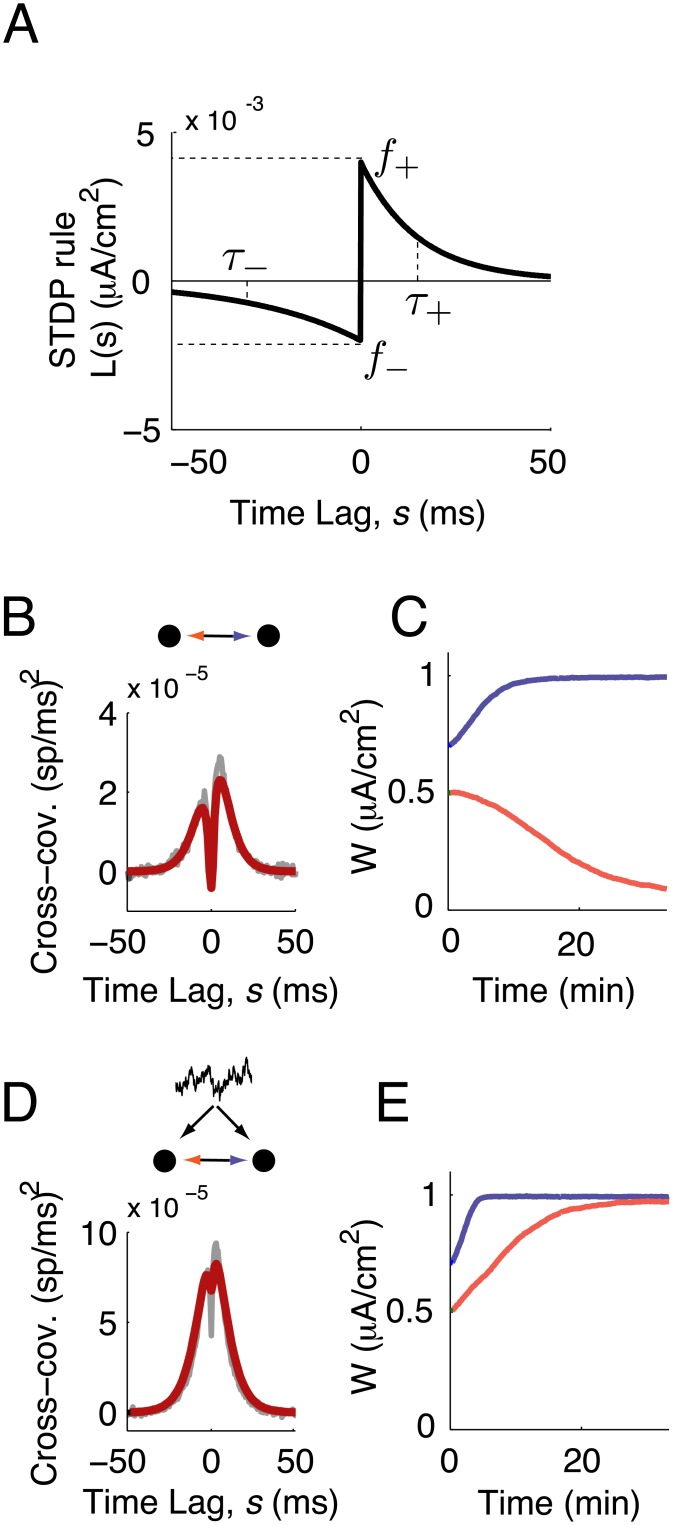
Network structure shapes synaptic plasticity. (A) The STDP rule, *L*(*s*), is composed of exponential windows for depression (-) and potentiation (+). Each is defined by its amplitude *f*_±_ and timescale *τ*_±_. (B) Spike train cross-covariance function for a pair of neurons with no common input, so that synapses between the two neurons are the only source of spiking covariance. Shaded lines: simulation, solid lines: theory (Eq (4)). (C,E) Synaptic weight (peak EPSC amplitude) as a function of time in the absence (C) and presence (E) of common input. (D) Spike train cross-covariance function for a pair of neurons with common input, *c* = 0.05. Common input was modeled as an Ornstein-Uhlenbeck process with a 5 ms timescale.

We begin by reviewing a well-studied phenomenological model of STDP [49], acting within a simple circuit of two reciprocally coupled neurons. Consider a pair of pre- and postsynaptic spike times with time lag *s* = *t*_post_−*t*_pre_. The evolution of the synaptic weight connecting presynaptic neuron *j* to postsynaptic neuron *i* obeys **W**_*ij*_ → **W**_*ij*_ + *L*(*s*), with the STDP rule *L*(*s*) (Fig 1A) being Hebbian:

$$L\left(s\right)=\{\begin{array}{cc} 𝓗\left(W^{\text{max}}-W_{ij}\right)f_{+}e^{-\frac{\left|s\right|}{\tau_{+}}}, & \text{if}\;s\geq 0\\ 𝓗\left(W_{ij}\right)(-f_{-})e^{-\frac{\left|s\right|}{\tau_{-}}}, & \text{if}\;s<0, \end{array}.$$ (1)

Here 𝓗(*x*) = 1 if *x* > 0 while 𝓗(*x*) = 0 if *x* ≤ 0, imposing bounds on the weights to prevent the magnitude of excitatory synapses from becoming negative or potentiating without bound (i.e. 0 ≤ **W**_*ij*_ ≤ *W* ^max^). The coefficients *f*_±_ scale the amplitude of weight changes induced by individual pre-post spike pairs and *τ*_±_ determine how synchronous pre- and postsynaptic spikes must be to drive plasticity.

The spike train from neuron *i* is the point process **y**_*i*_(*t*) = ∑_*k*_ *δ*(*t*−*t*_*ik*_), with *t*_*ik*_ being its *k* ^th^ spike time. Following [33] we relate the joint statistics of **y**_*i*_(*t*) and **y**_*j*_(*t*) to the evolution of synaptic weights. We assume that individual pre-post spike pairs induce small changes in synaptic weights (*f*_±_ ≪ *W* ^max^). This makes synaptic weights evolve slowly, on a much longer timescale than the millisecond scale of pairwise spiking covariance due to network interactions. The separation of timescales between synaptic plasticity and spiking activity provides an approximation to the evolution of the synaptic weights (Methods: learning dynamics):

$$\frac{dW_{ij}}{dt}=W_{ij}^{0}\int_{-\infty }^{\infty }L\left(s\right)(r_{i}r_{j}+C_{ij}\left(s\right))ds.$$ (2)

Here *r*_*i*_ is the time-averaged firing rate of neuron *i*, and **C**_*ij*_(*s*) = ⟨(**y**_*i*_(*t*) − *r*_*i*_)(**y**_*j*_(*t* + *s*) − *r*_*j*_)⟩ is the cross-covariance function of neuron *i* and *j*’s spike trains. The separation of timescales allows us to calculate the equilibrium spiking statistics **C**, taking **W** to be constant on the timescale of **C**(*s*). The term *r*_*i*_ *r*_*j*_ in Eq (2) captures the contribution of chance spike coincidences to STDP, while **C**_*ij*_(*s*) models the sensitivity of STDP to spike time correlations. Finally, **W** ^0^ is the adjacency matrix of the network—a binary matrix with ${\text{W}}_{ij}^{0}=1$ denoting the presence of a synapse from neuron *j* to neuron *i*. Multiplying by ${\text{W}}_{ij}^{0}$ ensures that synapses that do not exist cannot potentiate into existence. Eq (2) requires only the first and second order joint spiking statistics. To facilitate calculations, many previous studies have used Poisson neuron models with a specified *r*_*i*_ and **C**_*ij*_(*s*) to generate **y**_*i*_(*t*). In contrast, we will use a white noise-driven exponential integrate-and-fire model [50] for the generation of spike times (Methods: Neuron and network model). While this complicates the calculation of the spike train statistics, it provides a more biophysically realistic model of neural dynamics [51, 52] that better captures the timescales and neuronal nonlinearities that shape *r*_*i*_ and **C**_*ij*_(*s*). In total, the above theory determines synaptic evolution from the integrated combination of an STDP rule *L*(*s*) and the spike train cross-covariance function **C**_*ij*_(*s*). Thus, any mechanism affecting two neurons’ spiking covariance is expected to shape network structure through STDP.

As a simple illustration of how spiking correlations can drive STDP, we examined the synaptic weight dynamics, **W**_12_(*t*) and **W**_21_(*t*), in a reciprocally coupled pair of neurons, both in the presence and absence of common inputs. Specifically, the fluctuating input to neuron *i* was the sum of a private and common term, $\sqrt{1-c}\xi_{i}(t)+\sqrt{c}\xi_{c}(t)$, with *c* being the fraction of shared input to the neurons. In the absence of common input (*c* = 0; Fig 1B), the two synapses behaved as expected with Hebbian STDP: one synapse potentiated and the other depressed (Fig 1C). The presence of common input (*c* = 0.05) was a source of synchrony in the two neurons’ spike trains, inducing a central peak in the spike train cross-covariance function **C**_*ij*_(*s*) (Fig 1B vs 1D). In response to this increased synchrony both synapses potentiated (Fig 1E), in contrast to the case with *c* = 0. This was because of the sharp potentiation side of the learning rule compared to the the depression side (Fig 1A), so that increased spike synchrony enhanced the degree of overlap between **C**_*ij*_(*s*) and the potentiation component of *L*(*s*). This overcame the effects of depression in the initially weaker synapse and promoted strong, bidirectional connectivity in the two-neuron circuit.

This example highlights how the temporal shape of the spike train cross-covariance function, **C**_*ij*_(*s*), can interact with the shape of the learning rule, *L*(*s*), to direct STDP. However, this case only considered the effect of correlated inputs from outside of the modeled circuit. Our primary goal is to predict how spiking covariance due to internal network interactions combines with STDP to drive self-organized network structure. In order to do this, we first require a theory for predicting the spiking covariance between all neuron pairs given a static, recurrent connectivity. Once this theory has been developed, we will use it to study the case of plastic connectivity.

### Network architecture determines spiking covariance in static networks

In this section we review approximation methods [15, 47, 48] that estimate the pairwise spike train cross-covariances **C**_*ij*_(*s*) using a static weight matrix **W** (see Methods: Spiking statistics for a more full description). The exposition is simplified if we consider the Fourier transform of a spike train, ${\text{y}}_{i}(\omega )=\int_{-\infty }^{\infty }{\text{y}}_{i}(t)e^{-2\pi i\omega t}dt$, where *ω* is frequency. Assuming weak synaptic connections **W**_*ij*_, we approximate the spike response from neuron *i* as:

$$y_{i}\left(\omega \right)=y_{i}^{0}\left(\omega \right)+A_{i}\left(\omega \right)\sum_{j=1}^{N}W_{ij}J\left(\omega \right)y_{j}\left(\omega \right).$$ (3)

The function **A**_*i*_(*ω*) is the linear response [53] of the postsynaptic neuron, measuring how strongly modulations in synaptic currents at frequency *ω* are transferred into modulations of instantaneous firing rate about a background state ${\text{y}}_{i}^{0}$. The function *J*(*ω*) is a synaptic filter. In brief, Eq (3) is a linear ansatz for how a neuron integrates and transforms a realization of synaptic input into a spike train.

Following [15, 47, 48] we use this linear approximation to estimate the Fourier transform of **C**_*ij*_(*s*), written as ${\text{C}}_{ij}(\omega )=\langle {\text{y}}_{i}(\omega ){\text{y}}_{j}^{*}(\omega )\rangle$; here y* denotes the conjugate transpose. This yields the following matrix equation:

$$C\left(\omega \right)={\left(I-(W\cdot K(\omega ))\right)}^{-1}C^{0}\left(\omega \right){\left(I-(W\cdot K\left(\omega \right))*\right)}^{-1},$$ (4)

where **K**(*ω*) is an interaction matrix defined by **K**_*ij*_(*ω*) = **A**_*i*_(*ω*)**J**_*ij*_(*ω*). The matrix **C** ^0^(*ω*) is the covariance in the absence of synaptic coupling, with elements ${\text{C}}_{ij}^{0}(\omega )=\langle {\text{y}}_{i}^{0}(\omega ){\text{y}}_{j}^{0*}(\omega )\rangle$, and **I** is the identity matrix. Using Eq (4) we recover the matrix of spike train cross-covariance functions **C**(*s*) by inverse Fourier transformation. Thus, Eq (4) provides an estimate of the statistics of pairwise spiking activity in the full network, taking into account the network structure.

**Fig 2:**
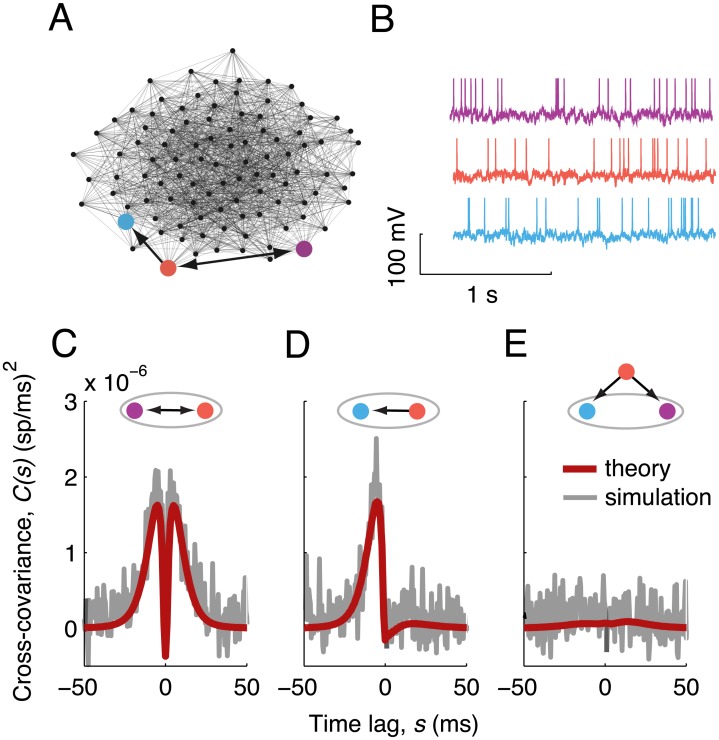
Linear response theory for spiking covariances. (A) Illustration of the network connectivity for a subset of 100 neurons. Three neurons, and the connections between them, are highlighted. Nodes are positioned by the Fruchterman-Reingold force algorithm. (B) Example voltage traces for the three highlighted neurons. (C-E) Spike train cross-covariance functions for the three combinations of labeled neurons. Top: A shaded ellipse contains the pair of neurons whose cross-covariance is shown. Shaded lines: simulations, red lines: linear response theory.

As a demonstration of the theory, we examined the spiking covariances of three neurons from a 1,000-neuron network (Fig 2A, colored neurons). The synaptic weight matrix **W** was static and had an adjacency matrix **W** ^0^ that was randomly generated with Erdös-Rényi statistics (connection probability of 0.15). The neurons received no correlated input from outside the network, making **C** ^0^(*ω*) a diagonal matrix, and thus recurrent network interactions were the only source of spiking covariance. Neuron pairs that connected reciprocally with equal synaptic weights had temporally symmetric spike train cross-covariance functions (Fig 2C), while unidirectional connections gave rise to temporally asymmetric cross-covariances (Fig 2D). When neurons were not directly connected, their covariance was weaker than that of directly connected neurons but was still nonzero (Fig 2E). The theoretical estimate provided by Eq (4) was in good agreement with estimates from direct simulations of the network (Fig 2C, 2D and 2E red vs. gray curves).

### Self-consistent theory for network structure and spiking covariance with plastic synapses

In general, it is challenging to develop theoretical techniques for stochastic systems with several variables and nonlinear coupling [53], such as in Eq (2). Fortunately, in our model the timescale of spiking covariance in the recurrent network with static synapses is on the order of milliseconds (Fig 2C, 2D and 2E), while the timescale of plasticity is minutes (Fig 1C and 1E). This separation of timescales provides an opportunity for a self-consistent theory for the coevolution of **C**(*s*) and **W**(*t*). That is, so long as *f*_±_ in Eq (1) are sufficiently small, we can approximate **W** as static over the timescales of **C**(*s*) and insert Eq (4) into Eq (2). The resulting system yields a solution **W**(*t*) that captures the long timescale dynamics of the plastic network structure (Methods: Self-consistent theory for network plasticity).

**Fig 3:**
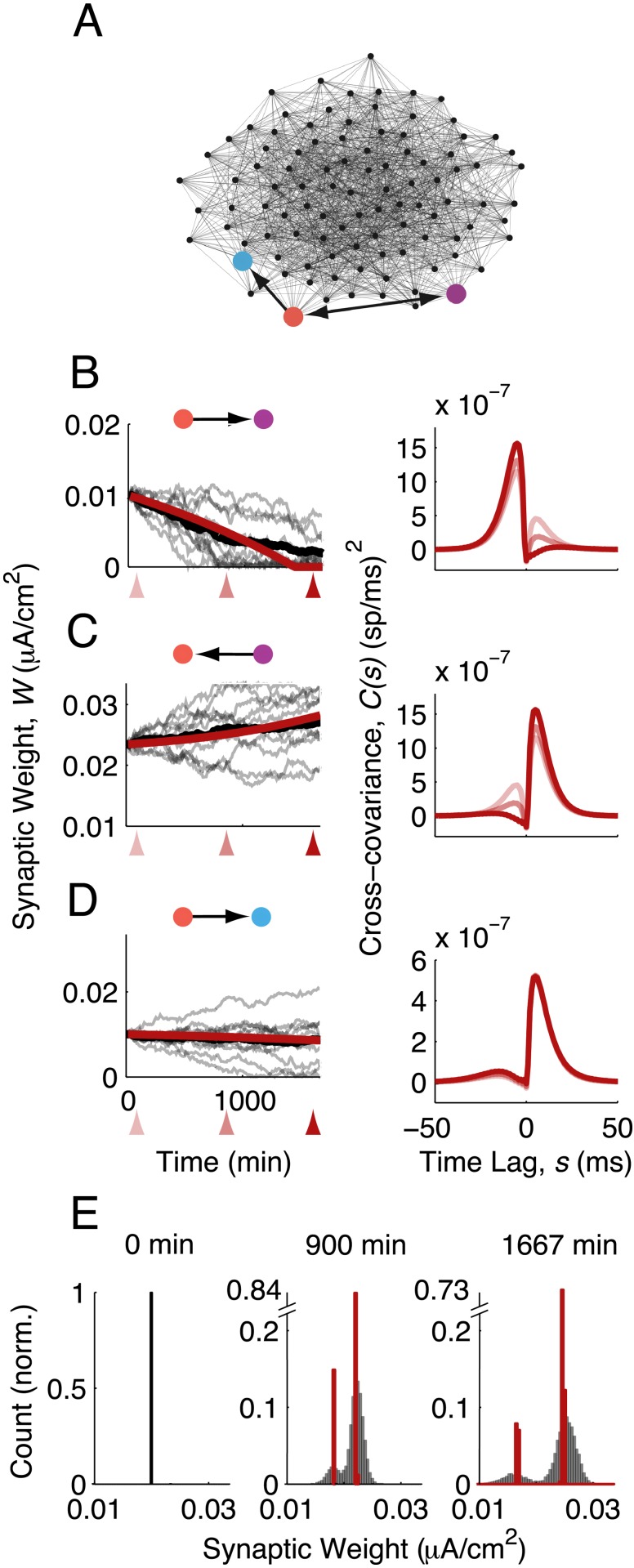
STDP in recurrent networks with internally generated spiking covariance. (A) As in Fig 2A. (B-D) Left, Synaptic weight versus time for each of the three synapses in the highlighted network. Shaded lines: simulation, individual trials of the same initial network. Solid black lines: simulation, trial-average. Solid red lines: theory. Right, spike train cross-covariances at the three time points marked on the left (linear response theory). (E) Histogram of synaptic weights at three time points. Red, theory. Shaded: simulation.

As a first illustration of our theory, we focus on the evolution of three synaptic weights in a 1,000-neuron network (Fig 3A, colored arrows). The combination of Eqs (2) and (4) predicted the dynamics of **W**(*t*), whether the weight decreased with time (Fig 3B left, red curve), increased with time (Fig 3C left, red curve), or remained approximately constant (Fig 3D left, red curve). In all three cases, the theory matched well the average evolution of the synaptic weight estimated from direct simulations of the spiking network (Fig 3B, 3C and 3D left, thick black curves). Snapshots of the network at three time points (axis arrows in Fig 3B, 3C and 3D, left), showed that **W** coevolved with the spiking covariance (Fig 3B, 3C and 3D right). We remark that for any realization of background input **y** ^0^(*t*), the synaptic weights **W**(*t*) deviated from their average value with increasing spread (Fig 3B, 3C and 3D left, thin black curves). This is expected since **C**(*t*) is an average over realizations of **y** ^0^(*t*), and thus provides only a prediction for the drift of **W**(*t*), while the stochastic nature of spike times leads to diffusion of **W**(*t*) around this drift [33]. In sum, the fast-slow decomposition of spiking covariance and synaptic plasticity provides a coherent theoretical framework to investigate the formation of network structure through STDP.

Our treatment is complementary to past studies on STDP that focused on the development of architecture through external input [35, 44, 54]. We restrict our analysis to networks with only internally generated correlations (i.e. ${\text{C}}_{ij}^{0}(s)=\langle {\text{y}}_{i}^{0}(t+s){\text{y}}_{j}^{0}(t)\rangle =0$ for *i* ≠ *j*), and thus focus on the formation of self-organized structure through STDP. A consequence of this modeling choice is low values of spiking correlations within the network: mean spike count correlation coefficients computed from all pairs within the network were approximately 5 × 10^−4^, and when conditioned on cell pairs having a direct connection between them were 4 × 10^−3^ (S1 Text). These low values agree with reports from unanethesized animals performing simple fixation task [55], or recordings restricted to cortical granule layers [56, 57], however a large number of other studies report significantly higher mean values of correlated activity [58].

There are several ways to increase the spiking correlations in our model. One is to assume weak external correlations in the background state (i.e. ${\text{C}}_{ij}^{0}(s)=\langle {\text{y}}_{i}^{0}(t+s){\text{y}}_{j}^{0}(t)\rangle >0$ for *i* ≠ *j*); this has been the focus of several past studies [47, 48]. Another is to reduce network size *N* to amplify any internally generated correlations within the network. When the network size was reduced from 1,000 to 100 the mean spike count correlation increased to 0.005 across all pairs and to 0.03 for directly coupled pairs (S1 Text). Despite these larger correlations, our self-consistent theory (Eqs (2) and (4)) predicted well the evolution of synaptic weights (S1 Fig). This reduction in *N* also increased the speed of learning by a factor of 10, however the separation of timescales required was still valid.

While our theory gives an accurate description of plasticity in the network, it is nevertheless high-dimensional. Keeping track of every individual synaptic weight and spike train cross-covariance function involves 𝓞(*N* ^2^) variables. For large networks, this becomes computationally challenging. More importantly, this high-dimensional theory does not provide insights into the plasticity of the *connectivity patterns* or *motifs* that are observed in cortical networks [3, 4]. Motifs involving two or more neurons represent correlations in the network’s weight matrix, which cannot be described by a straightforward application of mean-field techniques. In the next sections, we develop a principled approximation of the high-dimensional system to a closed low-dimensional theory for how the mean weight and the strength of two-synapse motifs evolve due to STDP.

### Dynamics of mean synaptic weight

We begin by considering the simple case of a network with unstructured weights. Analogous to having an Erdös-Rényi *adjacency* matrix **W** ^0^, we take there to be no second- or higher-order correlations in the *weight* matrix **W**. In this case, we can consider only the mean synaptic weight, *p*:

$$p=\frac{1}{N^{2}}\sum_{i,j}W_{ij}.$$ (5)

In order to calculate the dynamics of *p*, we insert the fast-slow STDP theory of Eq (2) into Eq (5):

$$\frac{dp}{dt}=\frac{1}{N^{2}}\sum_{i,j}W_{ij}^{0}\int_{-\infty }^{\infty }L\left(s\right)(r_{i}r_{j}+C_{ij}\left(s\right))ds,$$ (6)

where the spiking covariances are calculated using linear response theory (Eq (4)). This equation depends on the network structure in two ways. First, it depends on the full adjacency matrix **W** ^0^. Multiplying by ${\text{W}}_{ij}^{0}$ inside the average here prevents additional synapses from forming, so that we only consider the efficacy of synapses that exist, not the formation of new ones. Second, the spike train cross-covariances depend on the full weight matrix: **C**_*ij*_(*s*) = **C**_*ij*_(*s*;**W**). This dependence of a first–order connectivity statistic on the network structure poses a challenge for the development of a closed theory.

The main steps in our approach here are two approximations. First, the matrix of spike train cross-covariances **C**(*s*) obtained from our linear ansatz (Eq (4)) can be expanded in a power series around the background cross-covariances **C** ^0^(*s*) (see Eq (28)). Powers of the interaction matrix **K** in this series correspond to different lengths of paths through the network [13, 15]. We truncate the spiking covariances at length one paths to obtain:

$$C_{ij}\left(s\right)\approx \underset{\text{forward}}{\underset{︸}{(W_{ij}K_{ij}*C_{jj}^{0})\left(s\right)}}+\underset{\text{backward}}{\underset{︸}{(C_{ii}^{0}*W_{ji}K_{ji}^{-})\left(s\right)}}+\underset{\text{common}}{\underset{︸}{\sum_{k}(W_{ik}K_{ik}*C_{kk}^{0}*W_{jk}K_{jk}^{-})\left(s\right)}},$$ (7)

where * denotes convolution and $K_{ji}^{-}(t^{\prime })=K_{ji}(-t^{\prime })$. This truncation separates the sources of covariance between the spiking of neurons *i* and *j* into direct forward (*i* ← *j*) and backward (*i* → *j*) connections, and common (*k* → *i* and *k* → *j*) inputs. Nevertheless, after truncating **C**(*s*), the mean synaptic weight still depends on higher-order connectivity motifs (Eq (33)). Fortunately, for weak connections, these higher-order terms do not contribute substantially to overall spiking covariance (S2 Fig). This is especially true when we consider the covariance integrated against the plasticity rule *L*(*s*) (difference of 6%).

The second approximation is to ignore the bounds on the synaptic weight in Eq (1). While this results in a theory that only captures the transient dynamics of **W**(*t*), it greatly simplifies the derivation of the low-dimensional dynamics of motifs, because dynamics along the boundary surface are not considered.

With these two approximations, the mean synaptic weight obeys:

$$\frac{dp}{dt}=r^{2}S\frac{1}{N^{2}}\sum_{i,j}W_{ij}^{0}+S_{F}\frac{1}{N^{2}}\sum_{i,j}W_{ij}^{0}W_{ij}+S_{B}\frac{1}{N^{2}}\sum_{i,j}W_{ij}^{0}W_{ji}+S_{C}\frac{1}{N^{2}}\sum_{i,j,k}W_{ij}^{0}W_{ik}W_{jk}.$$ (8)

**Fig 4:**
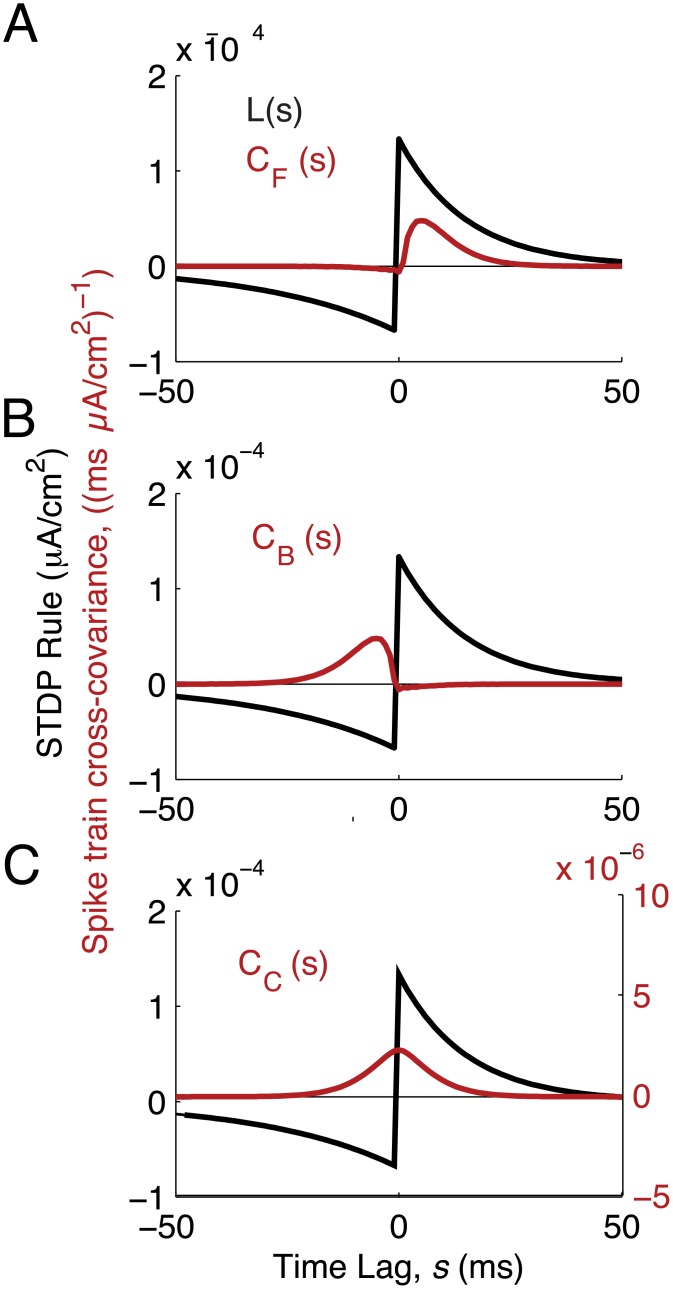
Different sources of spiking covariance interact with different parts of the STDP rule. Black: STDP rule. Red: spike train cross-covariances, from Eq (7). (A) Covariance from forward connections interacts with the potentiation side of the STDP rule. (B) Covariance from backward connections interacts with the depression side of the STDP rule. (C) Covariance from common input is temporally symmetric and interacts with both the potentiation and depression sides of the STDP rule.

The first term on the right hand side of Eq (8) is scaled by $S=\int_{-\infty }^{\infty }L(s)ds$, modeling the interaction between STDP and the mean firing rate, *r*, across the network. This captures STDP due to chance spiking coincidence and drives either net potentiation (*S* > 0) or depression (*S* < 0). The remaining terms capture how synaptic weights interact with the temporal structure of spiking covariance. Because of the expansion in Eq (7), these dependencies decompose into three terms, each scaled by the integral of the product of the STDP rule *L*(*s*) and a component of the spike train cross-covariance **C**(*s*). Specifically, covariance due to forward connections is represented by *S*_*F*_ (Eq (37); Fig 4A), covariance due to backward (reciprocal) connections is represented by *S*_*B*_ (Eq (38); Fig 4B), and finally covariance due to common connections is represented by *S*_*C*_ (Eq (39); Fig 4C).

For a network with unstructured weights, each sum in Eq (8) can be simplified. Let $p_{0}=\frac{1}{N^{2}}\sum_{i,j}{\text{W}}^{0_{ij}}$ be the connection density of the network. Since our theory for spiking covariances required weak synapses, we also explicitly scaled the weights, motifs, and amplitude of synaptic changes *f*_±_ by *ϵ* = 1/(*Np*_0_). This ensured that as the connection probability *p*_0_ was varied, synaptic weights scaled to keep the total input to a neuron constant (neglecting plasticity). The first and second terms of Eq (8) correspond to the definitions of *p*_0_ and *p*. Since different elements of **W** ^0^ and **W** are uncorrelated, the third term reduces to $\frac{1}{N^{2}}\sum_{i,j}{\text{W}}^{0_{ij}}{\text{W}}_{ji}=\epsilon pp_{0}+𝓞(\epsilon^{3/2})$ due to the central limit theorem. The last term can be similarly evaluated and the dynamics of *p* to first order in *ϵ* reduce to:

$$\frac{dp}{dt}=p_{0}r^{2}S+\epsilon (p(S_{F}+p_{0}S_{B})+p^{2}S_{C}).$$ (9)

We next study this mean-field theory in two regimes, before examining the plasticity of networks that exhibit motif structure.

### Unbalanced STDP of the mean synaptic weight

**Fig 5:**
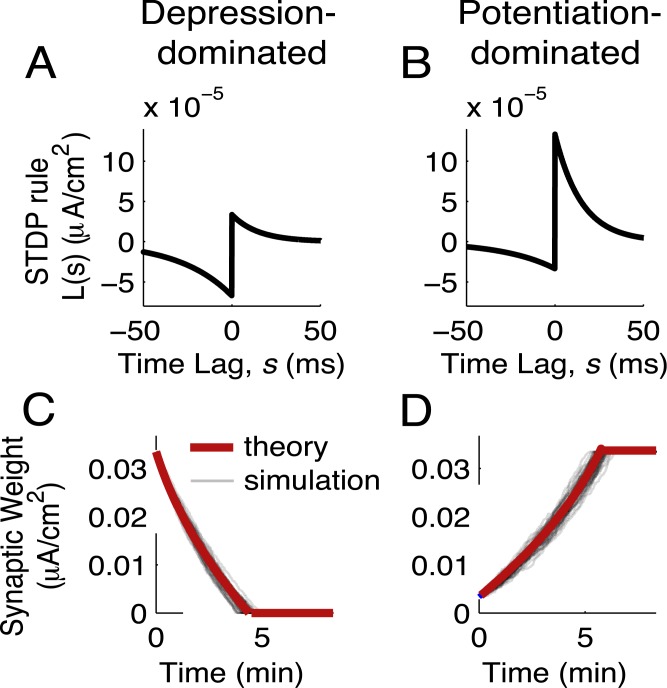
Unbalanced plasticity gives rise to simple weight dynamics. (A) Depression-dominated STDP rule: the amount of depression (integral of the depression side of the curve) is twice the amount of potentiation. (B) Potentiation-dominated STDP rule: the amount of potentiation is twice the amount of depression. (C) Evolution of synaptic weights with depression-dominated STDP: all weights depress. (D) Evolution of synaptic weights with potentiation-dominated STDP: all weights potentiate. Red lines: theory for mean synaptic weight. Shaded lines: simulation of individual synaptic weights.

Eq (9) contains one term proportional to the product of firing rates and the integral of the STDP rule, *r* ^2^ *S*, and additional terms proportional to the small parameter *ϵ*. When the learning rule, *L*(*s*), is dominated by either depression or potentiation (so that *S* ∼ 𝓞(1) ≫ *ϵ*) the whole network either uniformly depresses (Fig 5A and 5C) or potentiates (Fig 5B and 5D) due to chance spike coincidences (the firing rate term dominates in Eq (2)). These dynamics are straightforward at the level of individual synapses and this intuition carries over to the mean synaptic weight. When the STDP rule is dominated by potentiation or depression, the 𝓞(*ϵ*) terms in Eq (9) are negligible; the average plasticity is solely determined by the firing rates, with spiking covariance playing no role. In this case, the leading-order dynamics of *p* are:

$$p\left(t\right)=p_{0}r^{2}St+p\left(0\right),$$ (10)

so that the mean synaptic weight either potentiates to its upper bound *p*_0_ *W* ^max^ or depresses to 0, depending on whether the integral of the STDP rule, *S*, is positive or negative. For both depression- and potentiation-dominated STDP rules, our simple theory in Eq (10) quantitatively matches *p*(*t*) estimated from simulations of the entire network (Fig 5C and 5D, black vs. red curves).

### Balanced STDP of the mean synaptic weight

If there is a balance between potentiation and depression in the STDP rule *L*(*s*), then spiking covariance affects the average plasticity. In order to make explicit the balance between potentiation and depression, we write *S* = ±*δϵ* (with +*δϵ* for STDP with the balance tilted in favor of potentiation and −*δϵ* for balance tilted in favor of depression). The leading-order dynamics of *p* are then, for networks without motif structure,

$$\frac{1}{\epsilon }\frac{dp}{dt}=\pm \delta p_{0}r^{2}+p(S_{F}+p_{0}S_{B})+p^{2}S_{C}.$$ (11)

This quadratic equation admits up to two fixed points for *p*. We begin by examining the dynamics of *p* for the case perfectly balanced potentiation and depression (*δ* = 0) and a realistic shape of the STDP curve, and then consider the case of *δ* ≠ 0.

Experimentally measured STDP rules in cortex often show *f*_+_ > *f*_−_ and *τ*_+_ < *τ*_−_ [59, 60], making potentiation windows sharper and higher-amplitude than depression windows. In this case, the STDP-weighted covariance from forward connections, *S*_*F*_ > 0, is greater in magnitude than those from backward connections, *S*_*B*_ < 0 (Fig 4), and hence *S*_*F*_ + *p*_0_ *S*_*B*_ > 0. Furthermore, since the covariance from common input decays symmetrically around time lag *s* = 0 (Fig 4C), we have that *S*_*C*_ > 0. Consequently, when *δ* = 0, all terms in Eq (11) are positive and *p* potentiates to *p*_0_ *W* ^max^.

**Fig 6:**
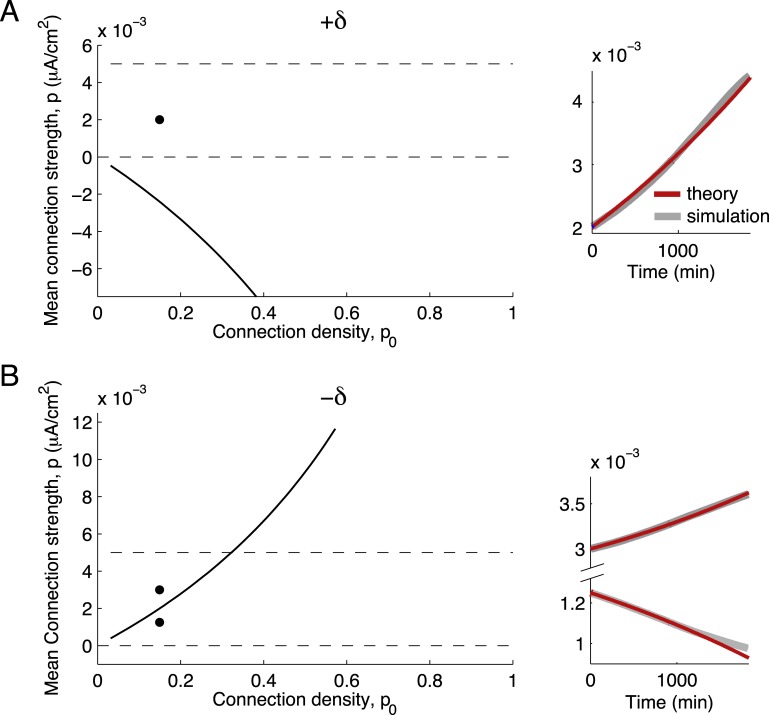
Balanced plasticity of the mean synaptic weight. (A) When the STDP rule is balanced and potentiation-dominated, the unstable fixed point for *p* is negative and decreases with the connection probability. (B) When the STDP rule is balanced and depression-dominated, the unstable fixed point is positive and increases with the connection probability. (A,B) Left: Dashed lines mark bounds for the mean synaptic weight, at 0 and *p*_0_ *W* ^max^. Black curves track the location of the unstable fixed point of *p* as the connection probability, *p*_0_, varies. Black dots mark initial conditions for the right panels. (A,B) Right: Dynamics of the mean synaptic weight in each of the regimes of the left plots. Red lines mark the reduced theory’s prediction (Eq (9)) and shaded lines the result of simulating the full spiking network (10 trials are plotted individually; they lie within line thickness of each other). Note that the ordinate axis has different limits in the left and right sides of the figure.

We next consider the case of imperfectly balanced STDP, with *δ* = 0.1. For potentiation-dominated balanced STDP, +*δϵ*, again all terms in Eq (11) are positive and *p* potentiates to *p*_0_ *W* ^max^ (Fig 6A). However, with depression-dominated balanced STDP (−*δϵ* in Eq (11)) *p* has two fixed points, at:

$$p=\frac{-\left(S_{F}+p_{0}S_{B}\right)\pm \sqrt{{\left(S_{F}+p_{0}S_{B}\right)}^{2}+4\delta p_{0}r^{2}S_{C}}}{2S_{C}}.$$ (12)

Since *S*_*F*_ + *p*_0_ *S*_*B*_ > 0 and *S*_*C*_ > 0 because of our assumptions on *f*_±_ and *τ*_±_, the term inside the square root is positive, making one fixed point positive and the other negative. The positive fixed point is unstable and, if within [0, *p*_0_ *W* ^max^], it provides a separatrix between potentiation and depression of *p* (Fig 6B). This separatrix arises from the competition between potentiation (due to forward connections and common input) and depression (due to reciprocal connections and firing rates).

Examination of Eq (12) shows competing effects of increasing the connection density *p*_0_: the *S*_*F*_ + *p*_0_ *S*_*B*_ terms decrease, while the 4*δp*_0_ *r* ^2^ *S*_*C*_ term increases. The latter effect dominates for the positive fixed point, raising the separatrix between potentiation and depression as *p*_0_ increases. So the mean synaptic weight of sparsely connected networks have a propensity to potentiate, while more densely connected networks are more likely to depress (Fig 6B).

In total, we see that a slight propensity for depression can impose bistability on the mean synaptic weight. In this case, a network with an initially strong mean synaptic weight *p*(0) can overcome depression and strengthen synaptic wiring, while a network with the same STDP rule and connection probability but with an initially weak mean synaptic weight will exhibit depression. In the next section we will show that similar separatrices exist in structured networks and govern the plasticity of different motifs.

## Motif dynamics

We now consider networks that have structure at the level of motifs. We begin by defining the weighted two-synapse motif variables:

$$\begin{array}{cc} q^{\text{div}} & =\frac{1}{N^{3}}\sum_{i,j,k}W_{ik}W_{jk}-p^{2},\\ q^{\text{con}} & =\frac{1}{N^{3}}\sum_{i,j,k}W_{ik}W_{ij}-p^{2},\\ q^{\text{ch}} & =\frac{1}{N^{3}}\sum_{i,j,k}W_{ij}W_{jk}-p^{2}. \end{array}$$ (13)

The variables *q* ^div^, *q* ^con^ and *q* ^ch^, respectively, measure the strength of divergent, convergent, and chain motifs. For each variable, we subtract the expected value of the sum in a network with uncorrelated weights, *p* ^2^, so that the *q*s measure above- or below-chance levels of structure in the network. Since these variables depend on the strength of both synapses making up the motif, we will refer to them as *motif strengths*. Motif strengths are also related to neurons’ (weighted) in- and out-degrees (the total strength of incoming or outgoing synapses for each neuron). The variables *q* ^div^ and *q* ^con^ are proportional to the variance of neurons’ in- and out-degrees, while *q* ^ch^, on the other hand, is proportional to the covariance of neurons’ in- and out-degrees. This can be seen by taking the definitions of these motifs, Eq (13), and first summing over the indices *i*, *j*. This puts the sum in *q* ^div^, for example, in the form of a sum over neurons’ squared out-degrees.

We remark that motif strengths (*q*) are separate from motif frequencies (*q*_0_). Motif frequencies have analogous definitions to Eq (13), but use the adjacency matrix **W** ^0^ instead of the weight matrix **W** (Eq (26)). It is clear that, for instance, $q^{\text{div}}\neq q_{0}^{\text{div}}$, although they would be proportional to one another if all weights **W**_*ij*_ were equal. An Erdös-Rényi network has an adjacency matrix **W** ^0^ with negligible motif frequencies. To avoid confusion, we refer to a network with negligible motif strengths as an unstructured network.

We wish to examine the joint dynamics of the mean synaptic weight *p* and the motif strengths. We insert the fast-slow STDP theory of Eq (2) into the definitions of *p* (Eq (5)) and the three *q*s (Eq (13)). Similarly to Eq (9), the dynamics of motifs *q* ^div^(*t*), *q* ^con^(*t*), and *q* ^ch^(*t*) then depend on the full network structure, **W**. This dependence of first- and second-order connectivity statistics on the network structure poses a challenge for the development of a closed theory for the dynamics of motifs. The main steps in developing such a theory are the two approximations we used to develop Eq (9), as well as one more.

As in the previous sections, our first approximation is to truncate the spike-train covariances at length one paths through the network. This removes the dependency of the dynamics on longer paths through the network. Nevertheless, after truncating **C**(*s*), the first- (*p*) and second-order (*q* ^div^, *q* ^con^, *q* ^ch^) motifs still depend on higher-order motifs (Eq (8)). This is because of coupling between lower and higher-order moments of the connectivity matrix **W** (see Eqs (33)–(35)) and presents a significant complication.

In order to close the dynamics at one- and two-synapse motifs, our new approximation follows [16], and we rewrite higher-order motifs as combinations of individual synapses and two-synapse motifs (see Eqs (40)–(41)). For the mean synaptic weight, for example, one third-order motif appears due to the common input term of the spike-train covariances (Eq (8)). We break up this three-synapse motif into all possible combinations of two-synapse motifs and individual connections, estimating its strength as:

$$\frac{1}{N^{3}}\sum_{i,j,k}W_{ij}^{0}W_{ik}W_{jk}\approx (p_{0}(q^{\text{div}}+p^{2})+p(q_{X}^{\text{con}}+q_{X}^{\text{ch}},B)).$$ (14)

This corresponds to assuming that there are no third- or higher-order correlations in the weight matrix beyond those due to second-order correlations; three- and more-synapse motifs are represented only as much as would be expected given the two-synapse motif strengths. We assume that all of the third- and higher-order cumulants of the weight and adjancency matrices that we encounter are zero. In total, this allows us to close the motif dynamics at two-synapse motifs. However, two new motifs appear in Eq (14), $q_{\text{X}}^{\text{con}}$ and $q_{\text{X}}^{\text{ch,B}}$. The subscript _X_ denotes that these motifs are mixed between the weight and adjacency matrices, measuring the strength of individual connections conditioned on their being part of a particular motif. $q_{\text{X}}^{\text{con}}$ corresponds to the strength of connections conditioned on being part of a convergent motif and $q_{\text{X}}^{\text{ch,B}}$ to the strength of connections conditioned on the postsynaptic neuron making another synapse in a chain (Eq (27)). As in previous sections, the final approximation is to ignore the bounds on the synaptic weight in Eq (1), so that our theory only captures the transient dynamics of **W**(*t*).

These approximations allow us (see Eqs (30), (33), and (42)) to rewrite the dynamics of the mean synaptic weight *p* as:

$$\frac{dp}{dt}=p_{0}r^{2}S+\epsilon [pS_{F}+(q_{X}^{\text{rec}}+p_{0}p)S_{B}+\frac{1}{p_{0}}(p_{0}(q^{\text{div}}+p^{2})+p(q_{X}^{\text{con}}+q_{X}^{\text{ch}},B))S_{C}].$$ (15)

The parameters *S*, *S*_*F*_, *S*_*B*_ and *S*_*C*_ are as defined in the previous section. Note that we recover Eq (9) when all *q*’s vanish (i.e. an unstructured network). When the network contains motif structure (*q* ≠ 0), the dynamics of *p* contain new terms. In Eq (15), the influence of forward connections through *S*_*F*_ is again proportional to the mean synaptic weight *p*. In contrast, the influence of backward connections *S*_*B*_ must interact with the new variable $q_{\text{X}}^{\text{rec}}$, which measures the mean strength of connections conditioned on their being part of a reciprocal loop (i.e. the strength of a backwards connection, conditioned on the existence of the forward one). As described above (Eq (14)), the covariance from common input *S*_*C*_ involves *p*, the divergent motif, *q* ^div^, as well as terms conditioned on weights being part of a convergent motif, $q_{\text{X}}^{\text{con}}$, or on the postsynaptic neuron making another synapse in a chain, $q_{\text{X}}^{\text{ch,B}}$. The definitions for the mixed motifs, the *q*_X_s, are given in Eq (27). In total, the dynamics of mean synaptic weight cannot be written as a single closed equation, but also requires knowledge of how the second order motifs evolve.

Fortunately, using a similar approach dynamical equations can be derived for each of the two-synapse motifs *q* ^div^, *q* ^cov^, and *q* ^ch^ (Eqs (43)–(45)). To close the system we require dynamics for five mixed motifs, $q_{\text{X}}^{\text{con}}$, $q_{\text{X}}^{\text{div}}$, $q_{\text{X}}^{\text{rec}}$, $q_{\text{X}}^{\text{ch,A}}$, and $q_{\text{X}}^{\text{ch,B}}$ (Eqs (46)–(50)). In total, this yields an autonomous 9-dimensional system of nonlinear differential equations describing the population-averaged plasticity of first- and second-order network structure. We have derived these equations in the absence of common external inputs to the neurons; the theory can easily be extended to this case by including external covariance in Eq (7) (replacing **C** ^0^ with (**C** ^0^ + **C** ^ext^), where **C** ^ext^ is the covariance matrix of the inputs).

When the network structure **W** ^0^ is approximately Erdös-Rényi, the motif frequencies *q*_0_ are 𝓞(*N* ^−3/2^) = 𝓞(*ϵ* ^3/2^). If we further assume initial conditions for the motif strengths and the mixed motifs to be unstructured (*q*(0) ∼ 𝓞(*ϵ* ^3/2^) for all motifs), then we also have *dq*_X_/*dt* ∼ 𝓞(*ϵ* ^3/2^) and *dq*_X_/*dt* ∼ 𝓞(*ϵ* ^3/2^) for each motif. In this case we can neglect, to leading order, the motifs entirely. Here the leading order dynamics simplify tremendously, and are restricted to the set $\left\{p(t),q^{\text{div}}=q^{\text{con}}=q^{\text{ch}}=q_{\text{X}}^{\text{rec}}=q_{\text{X}}^{\text{con}}=q_{\text{X}}^{\text{div}}=q_{\text{X}}^{\text{ch,A}}=q_{\text{X}}^{\text{ch,B}}=0\right\}$. Since the motif variables are zero the set corresponds to an unstructured network. Furthermore, since the leading order dynamics of the motif variables are zero this is an invariant set. The dynamics of *p*(*t*) then collapse to those given by Eq (9), which we have already examined (Figs 5 and 6).

The stability of that invariant set, however, remains to be determined. For finite *N*, the motif frequencies *q*_0_ will be non-zero even for (approximately) Erdös-Rényi networks. In this case we may consider the full system Eqs (42)–(50). In particular, the dynamics of the full system can be studied to determine the stability, or lack thereof, of the initial unstructured synaptic weights.

**Fig 7:**
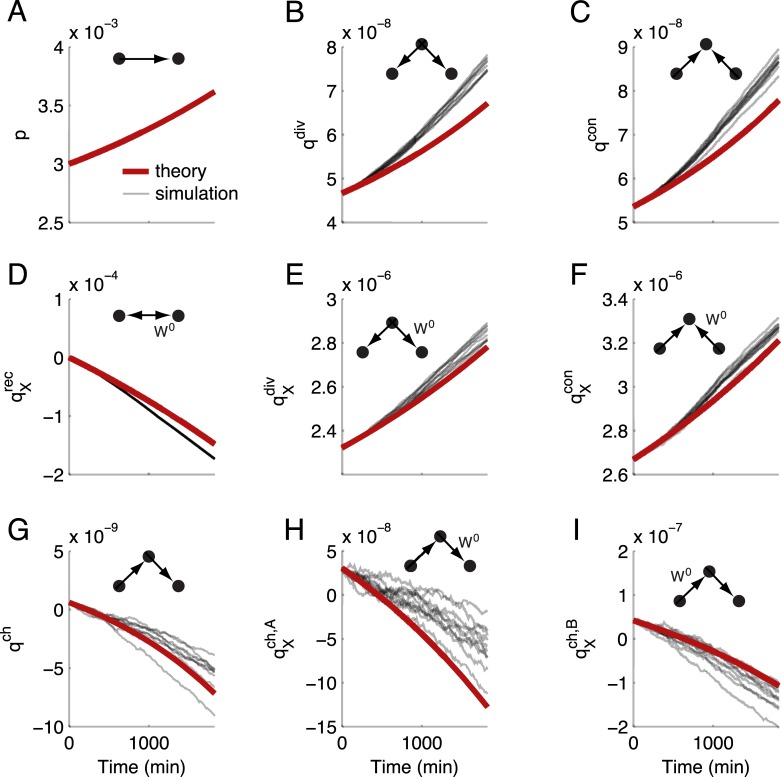
Reduced theory for the plasticity of two-synapse motifs. In each panel, the strength of a different motif or mixed motif is plotted as it evolves. Red: theoretical prediction (Eqs (42)–(50)). Shaded lines: individual trials of the same initial network. (A) Mean synaptic weight. (B) Divergent motifs. (C) Convergent motifs. (D) Mixed recurrent motifs (strength of connections conditioned on their being part of a two-synapse loop). (E) Mixed divergent motifs (strength of individual synapses conditioned on their being part of a divergent motif). (F) Mixed convergent motifs. (G) Chain motifs. (H) Mixed chains type A (strength of individual synapses conditioned on their being the first in a chain). (I) Mixed chains type B (strength of individual synapses conditioned on their being the second in a chain). The STDP rule was in the depression-dominated balanced regime, as in Fig 6B.

We refer to the mean field theory of Eqs (42)–(50) as the *motif dynamics* for a recurrent network with STDP. This theory accurately predicts the transient dynamics of the one- and two-synapse motifs of the full stochastic spiking network (Fig 7, compare red versus thin black curves), owing to significant drift compared to diffusion in the weight dynamics and these network-averaged motif strengths. The derivation and successful application of this reduced theory to a large spiking network is a central result of our study. However, recall that our theory requires the overall synaptic weights to be small so that our linear response ansatz remains valid. Thus, as expected, our theoretical predictions for the evolution of motif structure fail for sufficiently large initial mean synaptic weight *p*(0) (S2 Text). This is because for large recurrent weights the firing rate dynamics become unstable, and linearization about a background state is not possible.

Our theory captures several nontrivial aspects of the evolution of network structure. First, while the STDP rule is in the depression-dominated regime (*S* < 0 for the simulations in Fig 7), the mean synaptic weight *p* nevertheless grows (Fig 7A). Second, both divergent and convergent connections, *q* ^div^ and *q* ^con^, grow above what is expected for an unstructured network (Fig 7B and 7C); however, at the expense of chain connections *q* ^ch^ which decay (Fig 7G). The combination of these results show that for this STDP rule *L*(*s*), the unstructured network is not stable, and spontaneous structure forms slowly over time. In the subsequent sections, we leverage the simplicity of our reduced theory to gain insight into how the STDP rule interacts with recurrent architecture to drive motif dynamics.

### Unbalanced STDP of two-synapse motifs

When the STDP rule is dominated by potentiation or depression so that *S* ∼ 𝓞(1) ≫ *ϵ*, then the 𝓞(*ϵ*) terms in Eqs (43)–(50) are negligible. In this case each motif’s plasticity is solely determined by the firing rates, with spiking covariance playing no role. Here the motif dynamics are simply:

$$\begin{array}{cc} \frac{dp}{dt} & =p_{0}r^{2}S+O\left(\epsilon \right)\\ \frac{dq^{\alpha }}{dt} & =2r^{2}Sq_{X}^{\alpha }+O\left(\epsilon \right)\\ \frac{dq_{X}^{\alpha }}{dt} & =r^{2}Sq_{0}^{\alpha }+O\left(\epsilon \right) \end{array}$$ (16)

for *α* = div, con, or ch (and taking $q_{\text{X}}^{\text{ch}}=\left(q_{\text{X}}^{\text{ch,A}}+q_{\text{X}}^{\text{ch,B}}\right)/2$ in the second equation). The dynamics of *p* are the same here as for the unstructured case above; we include it for completeness. Dropping order *ϵ* terms gives the simple solutions:

$$\begin{array}{cc} p(t) & =p_{0}r^{2}St+p\left(0\right)\\ q^{\alpha }\left(t\right) & =q^{\alpha }\left(0\right)+q_{X}^{\alpha }\left(0\right)r^{2}St+\frac{1}{2}q_{0}^{\alpha }{\left(r^{2}S\right)}^{2}t^{2} \end{array}$$ (17)

for *α* = div, con, or ch (Methods: Unbalanced STDP). As stated previously, with *S* ∼ 𝓞(1), individual synapses uniformly potentiate or depress (Fig 5). This is reflected in the linear decay or growth (for depression- or potentiation-dominated *L*(*s*), respectively) of *p* with *r* ^2^ and quadratic amplification of baseline motif frequencies for the two-synapse motif strengths.

### Balanced STDP of two-synapse motifs

**Fig 8:**
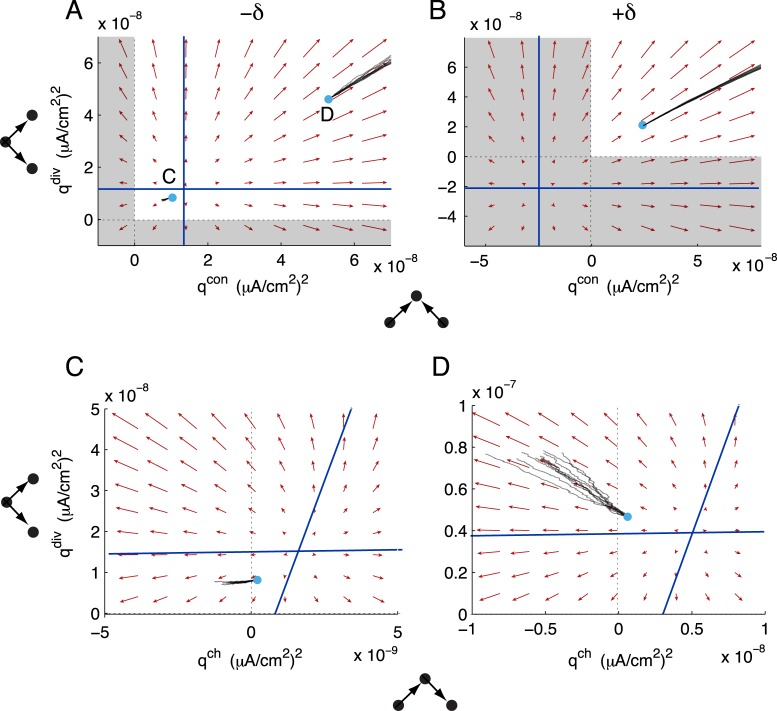
Plasticity of convergent and divergent motifs with balanced STDP. (A) Joint dynamics of convergent and divergent motifs when STDP is balanced and depression-dominated. Initial conditions as in Fig 7A. (B) Joint dynamics of convergent and divergent motifs when STDP is balanced and potentiation-dominated. Initial conditions as in Fig 7B. (C,D) Joint dynamics of divergent and chain motifs for the balanced, depression-dominated STDP rule. Initial conditions marked in panel A). Red: in all panels, the flow of the motif variables is projected into the corresponding plane, with all other motifs frozen at their initial conditions. Black: plasticity of the motifs in simulations of the full spiking network. Cyan dots mark initial conditions for the plotted variables. Each black trace is an individual realization of plasticity from the same initial network. For (A), the vector fields are indistinguishable, on the plotted scale, for both sets of initial conditions. In both panels, blue lines mark projections of each variable’s nullcline into the plane and regions of unattainable negative motif strengths are shaded.

Now we turn our attention to how internally generated spiking covariance interacts with balanced STDP to control motifs (examining the dynamics of Eqs (42)–(50)). As before, we consider STDP rules with sharper windows for potentiation than depression (*τ*_+_ < *τ*_−_ and *f*_+_ > *f*_−_). Each two-synapse motif can have a nullcline surface in the nine-dimensional motif space. These nullclines define a separatrix for the promotion or suppression of the corresponding motif, analogous to the case on the unstructured invariant set (Fig 7). We illustrate this by examining the dynamics in the (*q* ^div^,*q* ^con^) plane. For STDP rules with a balance tilted towards depression (−*δϵ*), the nullclines provided thresholds for the promotion or suppression of divergent or convergent motifs (Fig 8A, blue lines). The flow in this slice of the motif space predicted the motif dynamics well (Fig 8A, compare individual realizations of the full spiking network—thin black lines—to the flow defined by the vector field of the reduced motif system).

On the other hand, STDP rules with the balance tilted towards potentiation (+*δϵ*) have the nullclines at negative motif strengths (Fig 8B). Can the motif strengths achieve negative values? As stated previously, *q* ^con^ and *q* ^div^ are proportional to the variances of neurons’ in and out degrees, respectively. So, like the mean synaptic weight, *q* ^div^, *q* ^con^ ≥ 0, and these motifs always potentiated for +*δϵ* STDP rules (Fig 8B).

In examining the joint dynamics of divergent and convergent motifs, there is little evidence of interaction. The nullclines in the (*q* ^con^, *q* ^div^) plane are horizontal and vertical, so that whether divergent motifs potentiate or depress is independent of the dynamics of convergent motifs and vice versa (Fig 8A and 8B). This is reflected in the equations governing them. First, *q* ^div^ does not depend directly on *q* ^con^ (Eq (43)). Second, *q* ^con^ depends through *q* ^div^ only through the re-summed approximation of a four-synapse motif and the STDP-weighted covariances from common inputs, $S_{C}q^{\text{div}}q_{\text{X}}^{\text{con}}$ (Eq (44)) which due to the product $q^{\text{div}}q_{\text{X}}^{\text{con}}$ provides only weak dependency.

Chain motifs correspond to the covariance of neurons’ weighted in- and out-degrees and so, in contrast to *q* ^div^ and *q* ^con^, can achieve negative values. Indeed, the strength of chains can depress below zero even while the mean synaptic weight and other motifs potentiate (Fig 7A and 7G). Examining how *q* ^ch^, *q* ^div^ and *q* ^con^ coevolve allowed us to see how in- and out-hubs developed in the network. With the +*δϵ* STDP rule, *q* ^ch^ increased along with *q* ^con^ and *q* ^div^. So, individual neurons tended to become both in- and out-hubs. With the −*δϵ* STDP rule, however, *q* ^ch^ could decrease while *q* ^div^ and *q* ^con^ increased (Fig 7 and Fig 8D). In this case, neurons tended to become in- or out-hubs, but not both. In contrast to the vertical and horizontal nullclines in the (*q* ^con^, *q* ^div^) plane, *q* ^ch^ directly depends on *q* ^con^ and *q* ^div^ (Eq (45)). This is reflected in the nullcline structure of the (*q* ^ch^, *q* ^div^) plane: whether *q* ^ch^ potentiates or depresses depends on the initial strength of *q* ^div^ (Fig 8C and 8D). For these networks, *q* ^ch^ exhibited similar dependencies on *q* ^con^.

### Co-evolution of open chains and reciprocal loops

Many studies have examined how STDP affects either feedforward or recurrent structure in neuronal networks, commonly showing that STDP promotes feedforward structure at the expense of recurrent loops [36, 61, 62]. This is consistent with the intuition gained from isolated pairs of neurons, where STDP can induce competition between reciprocal synapses and eliminate disynaptic loops [24]. Our theory provides a new way to examine how STDP regulates feedforward vs recurrent motifs by examining the dynamics of *q* ^ch^. This variable includes both recurrent loops (*q* ^rec^) and open chains (*q* ^op^). In order to understand the contribution of each of these to overall potentiation or depression of chains, we split the motif strength for chains into contributions from recurrent loops and open chains, rewriting *q* ^ch^ as:

$$q^{\text{ch}}=\underset{q^{\text{rec}}}{\underset{︸}{\frac{1}{N^{3}}\sum_{i,j,k}\delta_{ik}W_{ij}W_{jk}}}+\underset{q^{\text{op}}}{\underset{︸}{\frac{1}{N^{3}}\sum_{i,j,k}(1-\delta_{ik})W_{ij}W_{jk}-p^{2}}}.$$ (18)

Similar to the case of other two-synapse motifs, the leading order dynamics of the recurrent motif are:

$$\frac{1}{2\epsilon }\frac{dq^{\text{rec}}}{dt}=r^{2}Sp_{0}(q_{X}^{\text{rec}}+pp_{0})+S_{F}q^{\text{rec}}+S_{B}q_{X2}^{\text{rec}}.$$ (19)

We obtain the dynamics of the feedforward motif by subtracting *dq* ^rec^/*dt* from *dq* ^ch^/*dt* (Eq (55)). In Eq (18) we subtract *p* ^2^ from *q* ^op^ because *q* ^op^ is the dominant contributor to *q* ^ch^. This restricts *q* ^rec^ to being non-negative. The new auxiliary variable $q_{\text{X2}}^{\text{rec}}$ is proportional to the conditional second moment of weights that are part of loops (Eq (52)), and evolves according to Eq (54). The replacement of *q* ^ch^ by these variables expands the motif space to 11 dimensions.

**Fig 9:**
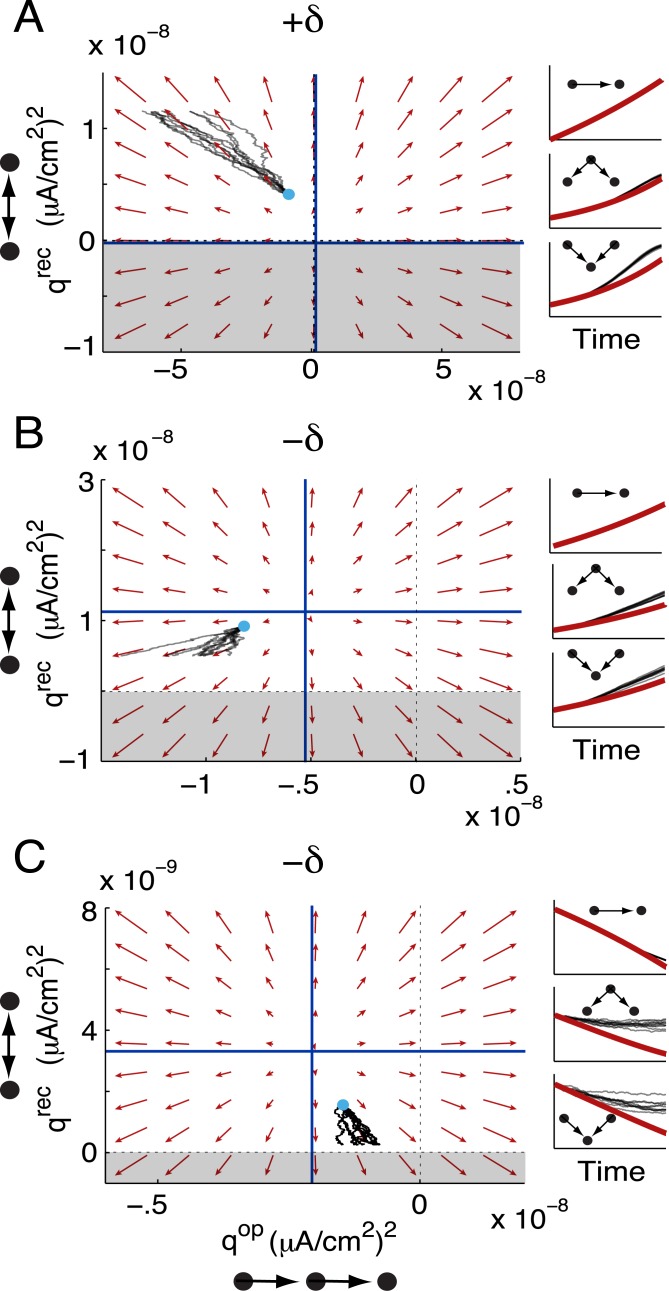
Plasticity of recurrent loops and open chains with balanced STDP. (A-C) The dynamics of loops and open chains, with all other variables fixed at their initial conditions. In all cases, the projections of the *q* ^op^ and *q* ^rec^ nullclines into this plane provide thresholds for the potentiation or depression of each motif. The shape of the STDP rule and the initial values of the other motif variables determine the locations of these nullclines. Color conventions are as in Fig 8. In each panel, right insets show the time series of *p* (top), *q* ^div^ (middle) and *q* ^con^ (bottom), with spiking simulations in black and motif theory in red. A) The potentiation-dominated balanced STDP rule. B) The depression-dominated balanced STDP rule, in the region where *p*, *q* ^div^ and *q* ^con^ potentiate. C) The depression-dominated balanced STDP rule, in the region where *p*, *q* ^div^ and *q* ^con^ depress.

We investigated the joint dynamics of open chains and recurrent loops by examining the (*q* ^op^, *q* ^rec^) plane. The *q* ^op^ and *q* ^rec^ nullclines divided this plane into regions where each motif potentiated or depressed. The shape of the STDP rule and the initial values of the other motif strengths affected the location of these nullclines. For the +*δϵ* STDP rule, the *q* ^rec^ nullcline was just below *q* ^rec^ = 0 (Fig 9A, blue horizontal line). Since *q* ^rec^ ≥ 0, this forced *q* ^rec^ to potentiate. The open chain motif, in contrast, could potentiate or depress above chance levels. In our spiking simulations, the initial conditions put *q* ^op^ in the region of depression, so that open chains depressed even while all other motifs were growing (Fig 9A, right panels).

These dynamics were the opposite of what would be expected from examining isolated pairs of neurons. With both the +*δϵ* and −*δϵ* balanced STDP rules, isolated pairs of neurons showed splitting of synaptic weights to eliminate the recurrent loop (S3 Fig). Thus, with the +*δϵ* STDP rule, the intuition gained from pairs of neurons did not predict the combined plasticity of open chains and recurrent loops. This is possible because our theory considers large networks that have both open chains and reciprocal loops in **W** ^0^, and the motif plasticity takes both into account.

The locations of the *q* ^op^ and *q* ^rec^ nullclines were sensitive to the values of the other motif variables. Since the mean synaptic weight and *q* ^div^ and *q* ^con^ exhibited bistability under the −*δϵ* STDP rule, we examined the (*q* ^op^, *q* ^rec^) slice through motif space when the other motifs were potentiating (Fig 9B, right panels) or depressing (Fig 9C, right panels). In both cases, the *q* ^rec^ nullcline was above 0 so that the recurrent motif could either potentiate or depress, depending on its initial strength (Fig 9B and 9C blue horizontal lines). Similarly, the feedforward motif could either potentiate or depress.

In spiking simulations with −*δϵ* STDP where *p* and the other motifs potentiated (Fig 9B, right), the initial conditions put (*q* ^op^, *q* ^rec^) in the region of phase space where they both depressed (Fig 9B, left). In spiking simulations with −*δϵ* STDP where *p* and other motifs depressed (Fig 9C, right), the initial conditions put (*q* ^op^, *q* ^rec^) in the region where *q* ^op^ potentiated and *q* ^rec^ depressed. This region corresponded to what would be expected from examining isolated pairs of neurons (S3 Fig): the loss of disynaptic loops and promotion of feedforward structure. So with the −*δϵ* STDP rule, the region of phase space where the pair-based intuition was accurate at the network level was accessible. In most of the motif space, however, interactions between triplets of neurons played a strong role so that the theory developed here was necessary to predict the STDP of motif structure.

### Motif dynamics in non-Erdös-Rényi networks

**Fig 10:**
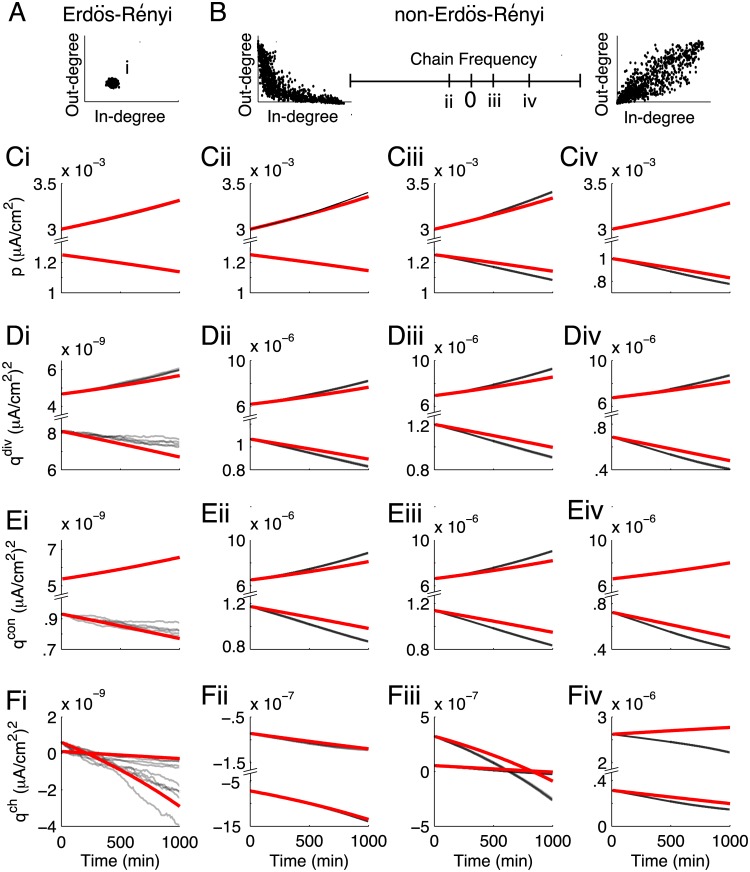
Motif dynamics in non-Erdös-Rényi networks. (A) Degree distribution of the finite-size Erdös-Rényi networks used in Figs 2–9, showing each neuron’s number of incoming synapses (in-degree) and outgoing synapses (out-degree). (B) Correlated, truncated power degree distributions. From left to right, the frequency of chain motifs increases. The degree distributions at either end of the “Chain Frequency” axis correspond to highly anti-correlated (left) and highly correlated (right) in- and out-degrees, with correlation coefficient *ρ* = ±.9 (Methods: Neuron and network models). The networks in columns ii-iv are drawn from the labelled points on this axis, with *ρ* = −.1 (ii), *ρ* = .1 (iii) and *ρ* = .5 (iv). In each column, we sample networks from each side of threshold for potentiation of *p*. For the network in column ii, $q_{0}^{\text{div}}=.0149$, $q_{0}^{\text{con}}=.0163$, $q_{0}^{\text{ch}}=-.0012$. For the network in column iii, $q_{0}^{\text{div}}=.0165$, $q_{0}^{\text{con}}=.0157$, $q_{0}^{\text{ch}}=7.7x10^{-4}$. For the potentiating network in column iv, $q_{0}^{\text{div}}=.0161$, $q_{0}^{\text{con}}=.0157$, $q_{0}^{\text{ch}}=.0062$. For the depressing network in column iv, $q_{0}^{\text{div}}=.0148$, $q_{0}^{\text{con}}=.0156$, $q_{0}^{\text{ch}}=.0068$. (C) Dynamics of the mean synaptic weight. (D) Dynamics of divergent motifs. (E) Dynamics of convergent motifs. (F) Dynamics of chain motifs. In all panels, the STDP rule is the balanced, depression-dominated one (−*δ* in Figs 6–9).

So far, we have examined the promotion or suppression of motif structure from initially unstructured networks with Erdös-Rényi **W** ^0^. In order to check how well our theory applied to non-Erdös-Rényi networks, we examined networks with truncated power law in- and out-degree distributions (Methods: Neuron and network models). These networks exhibited much higher levels of divergent and convergent motif structure (Fig 10D and 10E). They also violated the approximation we made that three- and four-synapse motifs are only as represented as would be expected from the two-synapse motifs we measure (e.g. Eq (14)).

For these networks, we varied the correlation of neurons’ in- and out-degrees, thus changing the frequency and initial strength of chains (Fig 10B). In most cases, we saw that our motif plasticity theory still matched simulations of the full spiking network’s evolution. This was true despite the motif variables being of several orders of magnitude larger compared to the Erdös-Rényi networks. In these networks, we see a similar bistability of the network structure to that observed earlier, both at the level of mean synaptic weights (Fig 10Cii-iv) and motifs (Fig 10D and 10Eii-iv).

When chain motifs were sufficiently over-represented, however, the theory qualitatively mis-predicted the actual evolution of *q* ^ch^ (Fig 10Fiv). Chain motifs play a large role in coupling various motifs to each other (Eqs (42)–(50)). So, it is not surprising that although all these non-Erdös-Rényi networks violated the re-summing approximation, we only saw the theory qualitatively break down when chain motifs were sufficiently strong. Thus, for this type of non-Erdös-Rényi network the theory developed here holds surprising promise for the investigation of motif plasticity.

## Discussion

We have developed a theory for spike timing-dependent plasticity in weakly-coupled recurrent networks of exponential integrate-and-fire neurons. We used this framework to derive a low-dimensional dynamical system capturing the plasticity of two-synapse motifs. The resulting system naturally classifies STDP rules into two categories: 1) rules with an imbalance between potentiation and depression and plasticity dominated by the firing rates of neurons in the network, and 2) rules with balanced potentiation and depression in which different sources of spiking covariance interact with the STDP rule to determine network structure. In the latter case, the importance of spiking covariances due to forwards connections, reciprocal connections, and common inputs creates new equilibrium points for the weighted motif structure of the network. For balanced, additive Hebbian STDP, these new equilibrium points are unstable. The nullcline manifolds that emanate from them divide the motif space into regions where different types of synaptic weight structure are either promoted or suppressed. When the balance in the STDP rule is tilted towards depression, regions where motifs are promoted or suppressed can both be accessible. For balanced STDP, any mechanism controlling spiking covariance in the network may affect how the network structure evolves. Thus, spike initiation dynamics [63–66], spike-frequency adaptation [67, 68], synaptic inhibition [69–71] and passive membrane properties [72] could all, in addition to controlling firing rates, drive motif dynamics.

### STDP in recurrent networks

A recent suite of studies derived a theory for how STDP shapes the full structure of networks of neurons whose spike trains are Poisson processes [35, 43–46, 54]. The initial approach is similar to ours with a linear approximation to estimate spiking covariance (see Eqs (3)–(4)). However, these studies mostly focused on the effects of external input, considering how feedforward inputs entrain structure in recurrent synapses [35, 44, 54]. The only source of spiking covariance was inherited from external sources (meaning **C**_0_(*s*) has off-diagonal structure), and subsequently filtered by the network to produce spiking covariance. Two previous studies by the same authors also examined STDP in networks without external stimuli [43, 45]; these took a large system size limit (*N* → ∞) and neglected the contribution of spiking covariance to STDP, focusing on the firing rate dependence due to an unbalanced learning rule.

In contrast, we consider the case where the intrinsic variability of neurons’ spike trains is the only source of spiking covariance, necessitating a finite sized network (*ϵ* = 1/(*Np*_0_) > 0). There is little difference between our results and those of past studies [43, 45] when the learning rule is unbalanced. If there is a balance between potentiation and depression, however, our theory shows how internally generated spiking covariances play a strong role in STDP-induced formation of self-organized structure. Furthermore, our use of integrate-and-fire models allows our theory to predict the evolution of network structure without fixing the statistics of individual or joint spiking activity.

We have focused here on networks composed only of excitatory neurons, a clear oversimplification of actual neural systems. The inclusion of inhibitory neurons would not, however, qualitatively change any of the results shown. Their effect on the plasticity of motifs can be understood by first considering their effect on the spike train covariances: in the first-order truncation of the spiking covariances (Eq 7), inhibitory neurons would provide additional common inputs to pairs of excitatory cells. If the inhibitory-excitatory projections are not plastic and have Erdös-Renyi connectivity, this would add a constant term to *dp*/*dt*. How the plasticity of inhibitory synapses [41, 73–75] interacts with excitatory plasticity to shape motif structure in neuronal networks remains an exciting open area of inquiry.

### Stability of learned network structures

Early studies of long-term plasticity, which gave rise to the phenomenological plasticity model we used, focused on the relative timing of action potentials. More recent experiments have shown that neurons’ firing rates and the postsynaptic membrane voltage and spike patterns all affect the shape of measured STDP curves [60, 76–79]. More complicated models of long-term plasticity, based on spike-triplet- or voltage-dependent STDP [80, 81] or on calcium thresholds for the induction of depression and potentiation [82–84], can replicate many of these complexities. The observation that firing rates undergo large fluctuations over slow timescales [85–89] suggests that *in vivo* STDP may transition between unbalanced potentiation- and depression-dominated regimes. While long-term plasticity can be strongly affected by pre- and postsynaptic firing rates, connectivity motifs and spiking covariance could determine the direction of plasticity during transitions between potentiation- and depression-dominated regimes. While our paper provides an initial framework to study how STDP shapes structure in recurrent networks, a more realistic learning rule than that used here (Eq (1)) will be needed to address these issues.

The additive, Hebbian STDP model we used here gives rise to splitting of synaptic weights: individual weights potentiate to some upper bound, or depress to a lower bound. This produces a bimodal distribution of synaptic weights, while experimentally observed weight distributions tend to be unimodal and long-tailed [3, 4, 90, 91]. Modifications of this model, such as introducing axonal or dendritic delays or weight-dependence of plasticity, can yield weight distributions more closely resembling those observed in neural tissue [30–32, 92, 93]. Depending on the modification made (delays vs weight-dependence), either the same or similar theories for motif plasticity can be derived using the methods presented in our study. Strong weight dependence, however, forces every weight to the same value so that the baseline motif frequencies completely determine the structure of the weight matrix (S4 Text). The dynamics of motifs under more realistic models of synaptic plasticity remain to be studied.

A major feature of STDP is that it can potentiate temporally correlated inputs [33]. Since synchronous inputs are effective at driving postsynaptic spiking, this can give rise to pathological activity in recurrent networks [39]. Synaptic depression driven by postsynaptic spikes, independent of presynaptic activity, can stabilize postsynaptic firing rates during STDP [29, 35]. Such additional rate-dependent terms of the plasticity rule can also stabilize the full weight matrix [45] and thus give rise to stable motif configurations. Recent work has focused on the necessity of homeostatic mechanisms, including synaptic scaling [94] or inhibitory plasticity [73], in stabilizing both the activity and structure of neural networks [36, 41, 95–98]. Since balanced STDP can give rise to bistability of mean synaptic weights in a network (Fig 7B), it could also provide a mechanism for assembly formation (selected weights potentiate, while other weights depress). Mechanisms of metaplasticity [99], operating on a similar timescale to STDP, could give rise to such a balance. This suggests a novel role for metaplasticity in controlling not only single-neuron excitability but also the self-organization of microcircuits in recurrent networks.

### Plasticity of motifs

Early studies on STDP focused on isolated pairs of reciprocally connected neurons, showing that the type of STDP we study tends to induce competition between reciprocal synapses (Fig 1B and 1C; [24]). Since then, many simulation studies have investigated how STDP affects the structure and activity of recurrent networks [38, 41, 75, 100–102], commonly examining the emergence of highly connected clusters. Reduced theories exposing how STDP shapes network-level structure have, however, been difficult to obtain. Most have examined the average synaptic weight in a network [103, 104], focusing on the relationship between network-averaged firing rates and mean synaptic weights (*p*) but neglecting spiking covariance. Mean-field theories are accurate for fully homogenous networks, however if all neurons have the same weighted in- and out-degrees there is no plasticity of two-synapse motifs (S3 Text).

The few reduced theories examining STDP of higher-order network structure have focused on the question of how STDP controls open chains versus recurrent loops. One study compared the mean strengths of feedforward versus recurrent inputs in a network receiving synchronous stimulation [62], but did so for a neuron that made no feedback connections to the network—effectively only taking into account the first term of Eq (7). Another study examined the strength of loops in a network of linear excitatory neurons, showing that STDP tends to reduce the total number of loops (of all lengths) in a network [61]. Our theory is restricted to two-synapse loops. While we have shown that these can potentiate (as in Fig 9C), [61] predicts that longer loops would meanwhile be weakened. Whether this is the case with balanced STDP driven by more realistic neuron models remains to be seen.

There is a growing body of evidence that cortical networks exhibit fine-scale structure [2–5]. Experimental studies have shown that such microcircuits depend on sensory experience [105, 106]. Our work provides an important advance towards explicitly linking the plasticity rules that control individual synapses and the emergent microcircuits of cortical networks. We have shown that synaptic plasticity based only on temporally precise spike-train covariance can give rise to a diversity and, under certain conditions, multistability of motif configurations. Motifs can have a strong influence on pairwise and population-level activity [8–18], suggesting that precise spike timing may play a role in how networks reorganize patterns of connectivity in order to learn computations.

## Methods

### Neuron and network model

We model a network of *N* neurons. The membrane dynamics of individual neurons obey the exponential integrate-and-fire (EIF) model [50], one of a class of models well-known to capture the spike initiation dynamics and statistics of cortical neurons [51, 52]. Specifically, the membrane voltage of neuron *i* evolves according to:

$$C\frac{dV_{i}}{dt}=g_{L}(V_{L}-V_{i})+g_{L}\Delta \text{exp}(\frac{V_{i}-V_{T}}{\Delta })+I_{i}\left(t\right)+\sum_{j=1}^{N}W_{ij}(J_{ij}*y_{j}.).$$ (20)

The first term on the right-hand side is the leak current, with conductance *g*_*L*_ and reversal potential *V*_*L*_. The next term describes a phenomenological action potential with an initiation threshold *V*_*T*_ and steepness Δ: when the voltage reaches *V*_*T*_, it diverges; this divergence marks an action potential. For numerical simulations, action potentials are thresholded at *V*(*t*) = *V*_th_, reset to a reset potential *V*_*re*_ and held there for an absolute refractory period *τ*_ref_.

Input from external sources not included in the model network is contained in *I*_i_(*t*). We model this as a Gaussian white noise process: *I*_*i*_(*t*) = *μ* + *g*_*L*_ *σDξ*_*i*_(*t*). The mean of the the external input current is *μ*. The parameter *σ* controls the strength of the noise and $D=\sqrt{\frac{2C}{g_{L}}}$ scales the noise amplitude to be independent of the passive membrane time constant. With this scaling, the infinitesimal variance of the passive membrane voltage is (*g*_*L*_ *σD*)^2^.

The last term of Eq (20) models synaptic interactions in the network. The *N* × *N* matrix **W** contains the amplitudes of each synapse’s postsynaptic currents. It is a weighted version of the binary adjacency matrix **W** ^0^, where ${\text{W}}^{0_{ij}}=1(0)$ indicates the presence (absence) of a synapse from neuron *j* onto neuron *i*. If a synapse *ij* is present then **W**_*ij*_ denotes its strength. Due to synaptic plasticity, **W** is dynamic: it changes in time as individual synapses potentiate or depress. The spike train from neuron *j* is the point process $y_{j}(t)=\sum_{k}\delta (t-t_{j}^{k})$, where $t_{j}^{k}$ denotes the *k* ^th^ spike time from neuron *j*. The *N* × *N* matrix **J**(*t*) defines the shape of the postsynaptic currents. In this study, we use exponential synapses: ${\text{J}}_{ij}(t-t_{j}^{k})=𝓗(t-t_{j}^{k})\text{exp}\left(-\frac{t-t_{j}^{k}}{\tau_{\text{S}}}\right)$, where 𝓗(*t*) is the Heaviside step function. Our theory is not exclusive to the EIF model or to the simple synaptic kernels we used; similar methods can be used with any integrate-and-fire model and arbitrary synaptic kernels. Model parameters are contained in Table 1 (unless specified otherwise in the text). In simulations, we took all synapses to initially have the same weight.

**Table 1:** Model parameters.

Parameter	Description	Value
*C*	Membrane capacitance	1 *μ*F/cm^2^
*g*_*L*_	Leak conductance	0.1mS/cm^2^
*V*_*L*_	Leak reversal potential	-72 mV
Δ	Action potential steepness	1.4 mV
*V*_*T*_	Action potential initiation threshold	-48 mV
*V*_*th*_	Action potential threshold	30 mV
*V*_*re*_	Action potential reset	-72 mV
*τ*_*ref*_	Action potential width	2 ms
*μ*	External input mean	1 *μ*A/cm^2^
*σ*	External input standard deviation	9 mV
*N*	Number of neurons	1000
*p*_0_	Connection density	0.15
*W* ^max^	Maximum synaptic weight	5 *μ*A/cm^2^
*τ*_S_	Synaptic time constant	5 ms

Unless otherwise stated we take the adjacency matrix **W**_0_ to have Erdös-Rényi statistics with connection probability *p*_0_ = 0.15 (Figs 2–9). For Fig 10, we generated networks with correlated, truncated power-law degree distributions. These obeyed:

$$p\left(d\right)=\{\begin{array}{cc} C_{1}d^{\gamma_{1}}, & 0\leq d\leq L_{1}\\ C_{2}d^{\gamma_{2}}, & L_{1}\leq L_{2}\\ 0, & \;\text{else} \end{array}$$ (21)

where *d* is the in- or out-degree. The marginal in- and out-degree distributions are coupled via a Gaussian copula with correlation coefficient *ρ* to generate the in- and out-degree lists. The degree lists are used to generate likelihoods for each possible connection ${\text{W}}_{ij}^{0}$, proportional to the in-degree of neuron *i* and out-degree of neuron *j*. We then sampled the elements of ${\text{W}}_{ij}^{0}$ according to these likelihoods.

We used *L*_1_ = *N*/10, *L*_2_ = *N*, *g*_1_ = 0.2, *g*_2_ = −1 in order to generate networks with large $q_{0}^{\text{div}},q_{0}^{\text{con}}$. The constants *C*_1_,*C*_2_ were chosen so that the mean in- and out-degrees were *Np*_0_ and the degree distribution was continuous at *L*_1_. The correlation of in- and out-degrees was *ρ* = −.1 (Fig 10, column ii), *ρ* = .1 (Fig 10, column iii) or *ρ* = .5 (Fig 10, column iv). The values of $q_{0}^{\text{div}},q_{0}^{\text{con}},q_{0}^{\text{ch}}$ for each network are reported in the caption of Fig 10. $q_{0}^{\text{div}}$ and $q_{0}^{\text{con}}$ were 𝓞(10^−2^). In contrast, in the Erdös-Rényi networks used earlier, $q_{0}^{\text{div}}$ and $q_{0}^{\text{con}}$ were 𝓞(10^−4^) and $q_{0}^{\text{ch}}$ was 𝓞(10^−6^).

### Learning dynamics

We now derive Eq (2), summarizing a key result of [33]. Changes in a synaptic weight **W**_*ij*_ are governed by the learning rule *L*(*s*), Eq (1). We begin by considering the total change in synaptic weight during an interval of length *T* ms:

$$\Delta W_{ij}=W_{ij}^{0}\int_{t}^{t+T}\int_{t}^{t+T}L\left(t^{\prime \prime }-t^{\prime }\right)y_{j}\left(t^{\prime \prime }\right)y_{i}\left(t^{\prime }\right)dt^{\prime \prime }dt^{\prime }$$ (22)

where multiplying by the corresponding element of the adjacency matrix ensures that nonexistent synapses do not potentiate into existence. Consider the trial-averaged rate of change:

$$\frac{\left\langle \Delta W_{ij}\right\rangle }{T}=W_{ij}^{0}\frac{1}{T}\int_{t}^{t+T}\int_{t-t^{\prime }}^{t+T-t^{\prime }}L\left(s\right)\left\langle y_{j}\left(t^{\prime }+s\right)y_{i}\left(t^{\prime }\right)\right\rangle dsdt^{\prime }$$ (23)

where *s* = *t*′′−*t*′ and ⟨⋅⟩ denotes the trial average. We first note that this contains the definition of the trial-averaged spike train cross-covariance:

$$C_{ij}\left(s\right)=\frac{1}{T}\int_{t}^{t+T}\left\langle y_{j}\left(t^{\prime }+s\right)y_{i}\left(t^{\prime }\right)\right\rangle dt^{\prime }-r_{i}r_{j}$$ (24)

where *r*_*i*_ is the time-averaged firing rate of neuron *i* and subtracting off the product of the rates corrects for chance spike coincidences. Inserting this definition into Eq (23) yields:

$$\frac{\left\langle \Delta W_{ij}\right\rangle }{T}=W_{ij}^{0}\int_{t-t^{\prime }}^{t+T-t^{\prime }}L\left(s\right)(r_{i}r_{j}+C_{ij}\left(s\right))ds$$ (25)

We then take the amplitude of individual changes in the synaptic weights to be small: *f*_+_,*f*_−_ < < *W* ^max^, where *τ*_±_ define the temporal shape of the STDP rule (see Eq (1)). In this case, changes in the weights occur on a slower timescale than the width of the learning rule. Taking *T* > > max(*τ*_+_,*τ*_−_) allows us to extend the limits of integration in Eq (25) to ±∞, which gives Eq (2). Note that in the results we have dropped the angle brackets for convenience. This can also be justified by the fact that the plasticity is self-averaging, since Δ**W**_*ij*_ depends on the integrated changes over the period *T*.

### Spiking statistics

In order to calculate *d* **W**_*ij*_/*dt*, we need to know the firing rates *r*_*i*_,*r*_*j*_ and spike train cross-covariance **C**_*ij*_(*s*) (Eq (2)). We take the weights to be constant on the fast timescale of *s*, so that the firing rates and spike train cross-covariances are stationary on that timescale. We solve for the baseline firing rates in the network via the self-consistency relationship

$$\begin{array}{cc} r_{i} & =r_{i}\left(\mu_{i}^{\text{eff}},\sigma \right),\text{where}\\ \mu_{i}^{\text{eff}} & =\mu +\sum_{j}(\int_{-\infty }^{\infty }J_{ij}\left(t\right)dt)W_{ij}r_{j} \end{array}$$

for *i* = 1,…,*N*. This gives the equilibrium state of each neuron’s activity. In order to calculate the spike train cross-covariances, we must consider temporal fluctuations around the baseline firing rates.

With sufficiently weak synapses compared to the background input, we can linearize each neuron’s activity around the baseline state. Rather than linearizing each neuron’s firing rate around *r*_*i*_, we follow [15, 47, 48] and linearize each neuron’s spike train around a realization of background activity, the uncoupled spike train ${\text{y}}_{0}^{i}$ (Eq (3)). The perturbation around the background activity is given by each neuron’s linear response function, **A**_*i*_(*t*), which measures the amplitude of firing rate fluctuations in response to perturbations of each neuron’s input around the baseline $\mu_{i}^{\text{eff}}$. We calculate **A**(*t*) using standard methods based on Fokker-Planck theory for the distribution of a neuron’s membrane potential [107, 108].

This yields Eq (3), approximating a realization of each neuron’s spike train as a mixed point and continuous process. Spike trains are defined, however, as pure point processes. Fortunately, Eq (2) shows that we do not need a prediction of individual spike train realizations, but rather of the trial-averaged spiking statistics. We can solve Eq (3) for the spike trains in the frequency domain as:

$$y\left(\omega \right)={\left(I-\left(W\cdot K\left(\omega \right)\right)\right)}^{-1}y^{0}\left(\omega \right)$$

where as in the Results, **K**(*ω*) is an interaction matrix defined by **K**_*ij*_(*ω*) = **A**_*i*_(*ω*)**J**_*ij*_(*ω*) and ⋅ denotes the element-wise product. Averaging this expression over realizations of the background spike trains yields a linear equation for the instantaneous firing rates. Averaging the spike trains **y** against each other yields the full cross-covariance matrix, Eq (4). It depends on the coupling strengths **W**, the synaptic filters **J**_*ij*_ and neurons’ linear response functions **A**, and the covariance of the baseline spike trains, **C** ^0^.

We can calculate the baseline covariance in the frequency domain, **C** ^0^(*ω*) = ⟨**y** ^0^ **y** ^0*^⟩, by first noting that it is a diagonal matrix containing each neuron’s spike train power spectrum. We calculate these using the renewal relationship between the spike train power spectrum **C** ^0^(*ω*) and the first passage time density [109]; the first passage time density for nonlinear integrate and fire models can be calculated using similar methods as for the linear response functions [108].

### Self-consistent theory for network plasticity

We solve the system Eqs (2) and (4) for the evolution of each synaptic weight with the Euler method with a time step of 100 seconds. At every time step of the plasticity, each neuron’s activity is re-linearized and the firing rates and spike train covariances recomputed. A package of code for solving the self-consistent theory and running the spiking simulations, in MATLAB and C, is available at http://sites.google.com/site/gabrielkochocker/code. Additional code is available on request.

### Derivation of motif dynamics

The baseline structure of the network is defined by the adjacency matrix **W** ^0^. The frequencies of different motifs are:

$$\begin{array}{cc} p_{0} & =\frac{1}{N^{2}}\sum_{i,j}W_{ij}^{0},\\ q_{0}^{\text{div}} & =\frac{1}{N^{3}}\sum_{i,j,k}W_{ik}^{0}W_{jk}^{0}-p_{0}^{2},\\ q_{0}^{\text{con}} & =\frac{1}{N^{3}}\sum_{i,j,k}W_{ik}^{0}W_{ij}^{0}-p_{0}^{2},\\ q_{0}^{\text{ch}} & =\frac{1}{N^{3}}\sum_{i,j,k}W_{ij}^{0}W_{jk}^{0}-p_{0}^{2}.\\ q_{0}^{\text{rec}} & =\frac{1}{N^{2}}\sum_{i,j}W_{ij}^{0}W_{ji}^{0}-p_{0}^{2}. \end{array}$$ (26)

Each of the *q*_0_ parameters refers to a different two-synapse motif. In divergent motifs ($q_{0}^{\text{div}}$), one neuron *k* projects to two others, *i* and *j*. In convergent motifs ($q_{0}^{\text{con}}$), two neurons *k* and *j* project to a third, *i*. In chain motifs ($q_{0}^{\text{ch}}$), neuron *k* projects to neuron *j*, which projects to neuron *i*. Finally, in recurrent motifs ($q_{0}^{\text{rec}}$) two neurons connect reciprocally. In each of these equations, we subtract off $p_{0}^{2}$ to correct for the baseline frequencies expected in Erdös-Rényi random networks. So, these parameters measure above-chance levels of motifs in the adjacency matrix **W** ^0^.

We extend this motif definition to a weighted version, given by Eq (13). Since our linear response theory for synaptic plasticity requires weak synapses, here we explicitly scale by the mean in-degree $\epsilon =\frac{1}{Np_{0}}$. In contrast to the motif frequencies, which depend only on the adjacency matrix **W** ^0^, the motifs here also depend on the weight matrix **W**.

$$\begin{array}{cc} \epsilon p & =\frac{1}{N^{2}}\sum_{i,j}W_{ij},\\ \epsilon^{2}q^{\text{div}} & =\frac{1}{N^{3}}\sum_{i,j,k}W_{ik}W_{jk}-\epsilon^{2}p^{2},\\ \epsilon^{2}q^{\text{con}} & =\frac{1}{N^{3}}\sum_{i,j,k}W_{ik}W_{ij}-\epsilon^{2}p^{2},\\ \epsilon^{2}q^{\text{ch}} & =\frac{1}{N^{3}}\sum_{i,j,k}W_{ij}W_{jk}-\epsilon^{2}p^{2},\\ \epsilon q_{X}^{\text{rec}} & =\frac{1}{N^{2}}\sum_{i,j}W_{ij}W_{ji}^{0}-\epsilon pp_{0},\\ \epsilon q_{X}^{\text{div}} & =\frac{1}{N^{3}}\sum_{i,j,k}W_{ik}W_{jk}^{0}-\epsilon pp_{0},\\ \epsilon q_{X}^{\text{con}} & =\frac{1}{N^{3}}\sum_{i,j,k}W_{ik}W_{ij}^{0}-\epsilon pp_{0},\\ \epsilon q_{X}^{\text{ch}},A & =\frac{1}{N^{3}}\sum_{i,j,k}W_{ij}W_{jk}^{0}-\epsilon pp_{0},\\ \epsilon q_{X}^{\text{ch}},B & =\frac{1}{N^{3}}\sum_{i,j,k}W_{ij}^{0}W_{jk}-\epsilon pp_{0} \end{array}$$ (27)

Here we have defined the two-synapse motifs, as well as five auxiliary variables, {*q*_X_}. These mixed motifs, defined by products of the weight and adjacency matrices, measure the strength of synapses *conditioned* on their being part of a motif. The motifs {*q*}, on the other hand, measure the total strength of the motifs. While the variables {*q*_X_} are not of direct interest, we will see that they are required in order to close the system of equations. In comparison to the motif *frequencies* {*q*_0_}, which measure motif frequencies in comparison to an independently *connected* network, the motif *strengths* are defined relative to an independently *weighted* network.

We also scale the amplitude of individual synaptic changes, *L*(*s*), by *ϵ*. We now go through the derivation of *dp*/*dt*, *dq* ^div^/*dt* and $dq_{\text{X}}^{\text{div}}/dt$ as examples; the other six variables follow the same steps. First, note that the spike train cross-covariance matrix of the network, Eq (4), can be expanded in the Fourier domain around the baseline covariance **C** ^0^(*ω*):

$$C\left(\omega \right)=(\sum_{i=0}^{\infty }{\left(W\cdot K\right)}^{i})C^{0}\left(\omega \right)(\sum_{j=0}^{\infty }{{\left((W\cdot K\right)}^{*})}^{i})$$ (28)

where the interaction matrix **W** ⋅ **K** is the element-wise product of the weight matrix **W** and the matrix of filters, **K**. Powers of **W** ⋅ **K** represent lengths of paths through the network. Only taking into account up to length one paths yields (for *i* ≠ *j*):

$$C_{ij}\left(s\right)\approx \underset{\text{forward connection}}{\underset{︸}{(W_{ij}K_{ij}*C_{jj}^{0})\left(s\right)}}+\underset{\text{backward connection}}{\underset{︸}{(C_{ii}^{0}*W_{ji}K_{ji}^{-})\left(s\right)}}+\underset{\text{common inputs}}{\underset{︸}{\sum_{k}(W_{ik}K_{ik}*C_{kk}^{0}*W_{jk}K_{jk}^{-})\left(s\right)}}.$$ (29)

where we have inverse Fourier transformed for convenience in the following derivation and **K** ^−^(*t*) = **K**(−*t*).

Differentiating each motif with respect to time, using the fast-slow STDP theory Eq (2) and inserting the first-order truncation of the cross-covariance functions, Eq (7), yields:

$$\begin{array}{cc} \epsilon \frac{dp}{dt} & =\frac{1}{N^{2}}\sum_{i,j}W_{ij}^{0}\int_{-\infty }^{\infty }\epsilon L\left(s\right)(r_{i}r_{j}+\delta_{ij}C_{ij}^{0}\left(s\right)+(W_{ij}K_{ij}*C_{jj}^{0})\left(s\right)\\ & \;\;\;+(C_{ii}^{0}*W_{ji}K_{ji}^{-})\left(s\right)+\sum_{k}(W_{ik}K_{ik}*C_{kk}^{0}*W_{jk}K_{jk}^{-}))ds \end{array}$$ (30)

$$\begin{array}{cc} \epsilon^{2}\frac{dq^{\text{div}}}{dt} & =\frac{2}{N^{3}}\sum_{i,j,k}[W_{ik}W_{jk}^{0}\int_{-\infty }^{\infty }\epsilon L\left(s\right)(r_{j}r_{k}+\delta_{jk}C_{jk}^{0}\left(s\right)+(W_{jk}K_{jk}*C_{kk}^{0})\left(s\right)\\ & \;\;\;+(C_{jj}^{0}*W_{kj}K_{kj}^{-})\left(s\right)+\sum_{l}(W_{jl}K_{jl}*C_{ll}^{0}*W_{kl}K_{kl}^{-}))ds]-2\epsilon^{2}p\frac{dp}{dt} \end{array}$$ (31)

$$\begin{array}{cc} \epsilon \frac{dq_{X}^{\text{div}}}{dt} & =\frac{1}{N^{3}}\sum_{i,j,k}W_{jk}^{0}W_{ik}^{0}\int_{-\infty }^{\infty }\epsilon L\left(s\right)(r_{i}r_{k}+\delta_{ik}C_{ik}^{0}\left(s\right)+(W_{ik}K_{ik}*C_{kk}^{0})\left(s\right)\\ & \;\;\;+(C_{ii}^{0}*W_{ki}K_{ki}^{-})\left(s\right)+\sum_{l}(W_{il}K_{il}*C_{ll}^{0}*W_{kl}K_{kl}^{-}))ds]-\epsilon p_{0}\frac{dp}{dt} \end{array}$$ (32)

We now define the network-averaged firing rate *r*, spike train autocovariances *C* ^0^ and linear response function. Since we model all postsynaptic currents with the same shape, this makes the matrix **K** a constant matrix; we replace its elements with the scalar *K*. Also neglecting the weight bounds in *L*(*s*) allows us to write:

$$\begin{array}{cc} \frac{dp}{dt} & =r^{2}S\frac{1}{N^{2}}\sum_{i,j}W_{ij}^{0}+S_{F}\frac{1}{N^{2}}\sum_{i,j}W_{ij}^{0}W_{ij}+S_{B}\frac{1}{N^{2}}\sum_{i,j}W_{ij}^{0}W_{ji}+S_{C}\frac{1}{N^{2}}\sum_{i,j,k}W_{ij}^{0}W_{ik}W_{jk} \end{array}$$ (33)

$$\begin{array}{cc} \epsilon \frac{dq^{\text{div}}}{dt} & =r^{2}S\frac{2}{N^{3}}\sum_{i,j,k}W_{ik}W_{jk}^{0}+S_{F}\frac{2}{N^{3}}\sum_{i,j,k}W_{ik}W_{jk}^{0}W_{jk}\\ \;\;\; & +S_{B}\frac{2}{N^{3}}\sum_{i,j,k}W_{ik}W_{jk}^{0}W_{kj}+S_{C}\frac{2}{N^{3}}\sum_{i,j,k,l}W_{ik}W_{jk}^{0}W_{jl}W_{kl}-2\epsilon p\frac{dp}{dt} \end{array}$$ (34)

$$\begin{array}{cc} \frac{dq_{X}^{\text{div}}}{dt} & =r^{2}S\frac{1}{N^{3}}\sum_{i,j,k}W_{jk}^{0}W_{ik}^{0}+S_{F}\frac{1}{N^{3}}\sum_{i,j,k}W_{jk}^{0}W_{ik}^{0}W_{ik}\\ \;\;\; & +S_{B}\frac{1}{N^{3}}\sum_{i,j,k}W_{jk}^{0}W_{ik}^{0}W_{ki}+S_{C}\frac{1}{N^{3}}\sum_{i,j,k,l}W_{jk}^{0}W_{ik}^{0}W_{il}W_{kl}-p_{0}\frac{dp}{dt} \end{array}$$ (35)

where we have cancelled off an *ϵ* from the left and right-hand sides. We have absorbed the integrals over the STDP rule and the spiking covariances into *r* ^2^ *S*, *S*_*F*_, *S*_*B*_ and *S*_*C*_. These correspond, respectively, to the total STDP-weighted spiking covariances from chance coincidence, forward connections, backward connections, and common input:

$$\begin{array}{cc} S & =\int_{-\infty }^{\infty }L\left(s\right)\;ds \end{array}$$ (36)

$$\begin{array}{cc} S_{F} & =\int_{-\infty }^{\infty }L\left(s\right)(K\left(t\right)*C^{0}\left(s\right))ds \end{array}$$ (37)

$$\begin{array}{cc} S_{B} & =\int_{-\infty }^{\infty }L\left(s\right)(C^{0}\left(s\right)*K^{-}\left(t\right))ds \end{array}$$ (38)

$$\begin{array}{cc} S_{C} & =\int_{-\infty }^{\infty }L\left(s\right)(K\left(t\right)*C^{0}\left(s\right)*K^{-}\left(t\right))ds \end{array}$$ (39)

These parameters depend on the spike train auto-covariance *C* ^0^(*s*) and interaction kernel *K*(*t*;*A*) of neurons. As the mean synaptic weight *p* changes, the average firing rate *r* will change and this will also affect *C* ^0^(*s*) and *K*(*t*;*A*). So *r*, *S*_*F*_, *S*_*B*_ and *S*_*C*_ are implicitly functions of *p* and thus evolve with the network. We have assumed weak synapses, so we expect small changes in firing rates and thus fix these at their value at *p* = *p*_0_ *W* ^max^/2, making *r*, *S*_*F*_, *S*_*B*_ and *S*_*C*_ constant parameters. In order to determine the impact of this approximation on our results, we compared the evolution of motifs in the reduced theory while re-calculating *r*, *S*_*F*_, *S*_*B*_ and *S*_*C*_ at every time-step. The approximation introduced negligible errors in calculating the evolution of the weighted motifs (S4 Fig).

Each dynamical equation now contains four different sums of products of the weight and adjacency matrices. First examining *dp*/*dt*, we see that the first three sums correspond to defined motifs: $1/N^{2}\sum_{i,j}{\text{W}}_{ij}^{0}=p_{0}$, $1/N^{2}\sum_{i,j}{\text{W}}_{ij}^{0}{\text{W}}_{ij}=p$ and $1/N^{2}\sum_{i,j}{\text{W}}_{ij}^{0}{\text{W}}_{ji}=q_{\text{X}}^{\text{rec}}+pp_{0}$. The last term in Eq (33), however, corresponds to a third-order motif mixed between the weight and adjacency matrices. Similarly, third- and fourth-order mixed motifs appear in Eqs 34 and 35. In order to calculate these, we extend a re-summing technique developed in [16]. We assume that there are no third- or higher-order correlations between elements of the weight and/or adjacency matrices, and approximate the frequency of each of these higher-order motifs by the number of ways it can be composed of one and two-synapse motifs. For a third order motif, this corresponds to adding up the likelihoods that all three synapses occur by chance and that each possible combination of one synapse and a two-synapse motif occur. In Eq (33),

$$\sum_{i,j,k}W_{ij}^{0}W_{ik}W_{jk}\approx \epsilon^{2}N^{3}(p_{0}(q^{\text{div}}+p^{2})+p(q_{X}^{\text{con}}+q_{X}^{\text{ch}},B)).$$ (40)

and for the four-synapse motif in Eq (34),

$$\sum_{i,j,k,l}W_{ik}W_{jk}^{0}W_{jl}W_{kl}\approx \epsilon^{3}N^{4}(p^{3}p_{0}+p^{2}(q_{X}^{\text{div}}+q_{X}^{\text{con}}+q_{X}^{\text{ch}},B)+pp_{0}(q^{\text{div}}+q^{\text{ch}})+q^{\text{div}}q_{X}^{\text{div}}+q^{\text{ch}}q_{X}^{\text{con}})$$ (41)

This re-summing, along with the inclusion of the mixed motifs {*q*_X_}, is what allows us to close the motif dynamics. Re-summing each third- and fourth-order motif in our system in terms of two-synapse motifs yields, after simplification, the final motif dynamics:

$$\begin{array}{cc} \frac{dp}{dt} & =p_{0}r^{2}S+\epsilon [pS_{F}+(q_{X}^{\text{rec}}+p_{0}p)S_{B}+\frac{1}{p_{0}}(p_{0}(q^{\text{div}}+p^{2})+p(q_{X}^{\text{con}}+q_{X}^{\text{ch}},B))S_{C}] \end{array}$$ (42)

$$\begin{array}{cc} \frac{dq^{\text{div}}}{dt} & =2r^{2}Sq_{X}^{\text{div}}+2\epsilon [q^{\text{div}}S_{F}+(p_{0}q^{\text{ch}}+pq_{X}^{\text{div}})S_{B}+\frac{1}{p_{0}}(q^{\text{ch}}(q_{X}^{\text{con}}+pp_{0})+q_{X}^{\text{div}}(q^{\text{div}}+p^{2}))S_{C}] \end{array}$$ (43)

$$\begin{array}{cc} \frac{dq^{\text{con}}}{dt} & =2r^{2}Sq_{X}^{\text{con}}+2\epsilon [q^{\text{con}}S_{F}+(p_{0}q^{\text{ch}}+pq_{X}^{\text{con}})S_{B}+\frac{1}{p_{0}}(q^{\text{con}}(q_{X}^{\text{ch}},B+pp_{0})+q_{X}^{\text{con}}(q^{\text{div}}+p^{2}))S_{C}] \end{array}$$ (44)

$$\begin{array}{cc} \frac{dq^{\text{ch}}}{dt} & =r^{2}S(q_{X}^{\text{ch}},A+q_{X}^{\text{ch}},B)+\epsilon [2q^{\text{ch}}S_{F}+(p_{0}(q^{\text{con}}+q^{\text{div}})+p(q_{X}^{\text{ch}},A+q_{X}^{\text{ch}},B))S_{B}\\ & \;\;\;+\frac{1}{p_{0}}((q^{\text{div}}+p^{2})(q_{X}^{\text{ch}},A+q_{X}^{\text{ch}},B)+q^{\text{ch}}(q_{X}^{\text{ch}},B+pp_{0})+q^{\text{con}}(q_{X}^{\text{con}}+pp_{0}))S_{C}] \end{array}$$ (45)

$$\begin{array}{cc} \frac{dq_{X}^{\text{rec}}}{dt} & =r^{2}Sq_{0}^{\text{rec}}+\epsilon [q_{X}^{\text{rec}}S_{F}+(1-p_{0})(q_{X}^{\text{rec}}+pp_{0})S_{B}\\ & \;\;\;+\frac{1}{p_{0}}(q_{0}^{\text{rec}}(q^{\text{div}}+p^{2})+q_{X}^{\text{ch}},B(q_{X}^{\text{ch}},B+pp_{0})+q_{X}^{\text{con}}(q_{X}^{\text{con}}+pp_{0}))S_{C}] \end{array}$$ (46)

$$\begin{array}{cc} \frac{dq_{X}^{\text{div}}}{dt} & =r^{2}Sq_{0}^{\text{div}}+\epsilon [q_{X}^{\text{div}}S_{F}+(pq_{0}^{\text{div}}+p_{0}q_{X}^{\text{ch}},B)S_{B}+\frac{1}{p_{0}}(q_{0}^{\text{div}}(q^{\text{div}}+p^{2})+q_{X}^{\text{ch}},B(q_{X}^{\text{con}}+pp_{0}))S_{C}] \end{array}$$ (47)

$$\begin{array}{cc} \frac{dq_{X}^{\text{con}}}{dt} & =r^{2}Sq_{0}^{\text{con}}+\epsilon [q_{X}^{\text{con}}S_{F}+(pq_{0}^{\text{con}}+p_{0}q_{X}^{\text{ch}},A)S_{B}+\frac{1}{p_{0}}(q_{0}^{\text{con}}(q^{\text{div}}+p^{2})+q_{X}^{\text{con}}(q_{X}^{\text{ch}},B+pp_{0}))S_{C}] \end{array}$$ (48)

$$\begin{array}{cc} \frac{dq_{X}^{\text{ch}},A}{dt} & =r^{2}Sq_{0}^{\text{ch}}+\epsilon [q_{X}^{\text{ch}},AS_{F}+(pq_{0}^{\text{ch}}+p_{0}q_{X}^{\text{con}})S_{B}+\frac{1}{p_{0}}(q_{0}^{\text{ch}}(q^{\text{div}}+p^{2})+q_{X}^{\text{con}}(q_{X}^{\text{con}}+pp_{0}))S_{C}] \end{array}$$ (49)

$$\begin{array}{cc} \frac{dq_{X}^{\text{ch}},B}{dt} & =r^{2}Sq_{0}^{\text{ch}}+\epsilon [q_{X}^{\text{ch}},BS_{F}+(pq_{0}^{\text{ch}}+p_{0}q_{X}^{\text{div}})S_{B}+\frac{1}{p_{0}}(q_{0}^{\text{ch}}(q^{\text{div}}+p^{2})+q_{X}^{\text{ch}},B(q_{X}^{\text{ch}},B+pp_{0}))S_{C}] \end{array}$$ (50)

Examination of these equations reveals how different types of joint spiking activity affect motif dynamics. Chance spiking coincidence (the *r* ^2^ *S* terms) couple each motif to the mixed version of itself, and each mixed motif to the baseline structure of the adjacency matrix. With Hebbian STDP and excitatory synapses, *S*_*F*_ > 0 and *S*_*B*_ < 0. So, spiking covariance from forward connections provide positive feedback, reinforcing the current network structure. Spiking covariance from backward connections and common input couple divergent, convergent and chain motifs to each other.

The dynamics on the invariant set (Results: Balanced STDP of the mean synaptic weight, Fig 6) were plotted in MATLAB. The vector fields of Figs 8 and 9 were calculated in XPPAUT [110]. For those figures, results from simulations of the full spiking network were plotted in MATLAB and then overlaid on the vector fields from XPPAUT.

#### Plasticity of loops and open chains

The chain variable *q* ^ch^ includes both open chains and recurrent loops. (open chains correspond to *k* ≠ *i* in the definition of *q* ^ch^, Eq (27), and recurrent loops to *k* = *i*.) As in the main text, we break *q* ^ch^ into these two cases: *q* ^ch^ = *q* ^rec^ + *q* ^op^, where

$$\begin{array}{cc} \epsilon^{2}q^{\text{rec}} & =\frac{1}{N^{3}}\sum_{i,j,k}\delta_{ik}W_{ij}W_{jk}=\frac{1}{N^{3}}\sum_{i,j}W_{ij}W_{ji}\\ \epsilon^{2}q^{\text{op}} & =\frac{1}{N^{3}}\sum_{i,j,k}(1-\delta_{ik})W_{ij}W_{jk}-\epsilon^{2}p^{2} \end{array}$$ (51)

We also define an auxiliary variable which we will require in the dynamics of *q* ^rec^:

$$\epsilon^{2}q_{X2}^{\text{rec}}=\frac{1}{N^{3}}\sum_{i,j}W_{ij}^{2}W_{ji}^{0}$$ (52)

which is proportional to the conditioned second moment of weights that are part of disynaptic loops. The dynamics of *q* ^rec^ are calculated exactly as for the other motifs and are:

$$\frac{1}{2\epsilon }\frac{dq^{\text{rec}}}{dt}=r^{2}Sp_{0}(q_{X}^{\text{rec}}+pp_{0})+S_{F}q^{\text{rec}}+S_{B}q_{X2}^{\text{rec}}$$ (53)

where the new auxiliary variable obeys

$$\frac{1}{2\epsilon }\frac{dq_{X2}^{\text{rec}}}{dt}=r^{2}Sp_{0}(q_{X}^{\text{rec}}+pp_{0})+S_{F}q_{X2}^{\text{rec}}+S_{B}q^{\text{rec}}$$ (54)

We can then recover the dynamics of open chains as:

$$\begin{array}{cc} \frac{dq^{\text{op}}}{dt} & =\frac{dq^{\text{ch}}}{dt}-\frac{dq^{\text{rec}}}{dt}\\ & =r^{2}S(q_{X}^{\text{ch},A}+q_{X}^{\text{ch},B})+\epsilon [-2r^{2}Sp_{0}(q_{X}^{\text{rec}}+pp_{0})+2S_{F}q^{\text{op}}\\ & \;\;\;+(p_{0}(q^{\text{con}}+q^{\text{div}})+p(q_{X}^{\text{ch},A}+q_{X}^{\text{ch},B})-2q_{X}2^{\text{rec}})S_{B}\\ & \;\;\;\frac{1}{p_{0}}((q^{\text{div}}+p^{2})(q_{X}^{\text{ch},A}+q_{X}^{\text{ch},B})+q^{\text{ch}}(q_{X}^{\text{ch},B}+pp_{0})+q^{\text{con}}(q_{X}^{\text{con}}+pp_{0}))S_{C}] \end{array}$$ (55)

#### Unbalanced STDP

When there is an imbalance between the net amounts of potentiation and depression in the STDP rule, the motif dynamics are governed by simpler equations. If *S* ∼ 𝓞(1), the 𝓞(*ϵ*) terms in Eqs 42–50 are negligible. For each mixed motif,

$$q_{X}\left(t\right)=r^{2}Sq_{0}t+q_{X}\left(0\right)$$ (56)

so that

$$p\left(t\right)=p_{0}r^{2}St+p\left(0\right)$$ (57)

$$q^{\text{div}}(t)=q^{\text{div}}(0)+q_{\text{X}}^{\text{div}}(0)r^{2}St+\frac{1}{2}q_{0}^{\text{div}}{\left(r^{2}S\right)}^{2}t^{2}$$ (58)

$$q^{\text{con}}(t)=q^{\text{con}}(0)+q_{\text{X}}^{\text{con}}(0)r^{2}St+\frac{1}{2}q_{0}^{\text{con}}{\left(r^{2}S\right)}^{2}t^{2}$$ (59)

$$q^{\text{ch}}(t)=q^{\text{ch}}(0)+\left(q_{\text{X}}^{\text{ch},\text{A}}(0)+q_{\text{X}}^{\text{ch},\text{B}}(0)\right)r^{2}St+\frac{1}{2}q_{0}^{\text{ch}}{\left(r^{2}S\right)}^{2}t^{2}$$ (60)

Writing $q_{\text{X}}^{\text{ch}}=q_{\text{X}}^{\text{ch,A}}+q_{\text{X}}^{\text{ch,B}}$ puts the dynamics for all the motifs in the same form. The motifs expand from the initial conditions and baseline structure of the network. Note that since the quadratic term is proportional to *S* ^2^, even when STDP is depression-dominated the long-term dynamics are expansive rather than contractive.

## Supporting Information

S1 Text Magnitude of spike count correlations. (PDF)

S2 Text Strength of synaptic weights and stability of firing rates. (PDF)

S3 Text Motif plasticity in homogenous networks. (PDF)

S4 Text Motif plasticity with weight-dependent (multiplicative) STDP. (PDF)

S1 Fig Plasticity in networks with larger spiking correlations. (PDF)

S2 Fig Truncated vs full spike train cross-covariances. (PDF)

S3 Fig Balanced STDP in isolated pairs of neurons. (PDF)

S4 Fig Rate dependence of spike train covariability. (PDF)

S1 Code Scripts (written in MATLAB and C) for the spiking simulations and theory of Figs 1–3. (ZIP)
