# Supplementary material for: Self-Organization of Microcircuits in Networks of Spiking Neurons with Plastic Synapses
Source: PLoS Comput Biol. 2015 Aug 20;11(8):e1004458. doi: 10.1371/journal.pcbi.1004458 (PMC4546203; doi:10.1371/journal.pcbi.1004458)
Supplement: S1 Text — (PDF) [file pcbi.1004458.s001.pdf]

Supplemental Material S1 Text: Magnitude of spike count correlations

Here we compute spike count correlations for the studied networks. A spike count from neuron i, $n_i^T(t)$ is the number of spikes occurring within the window $(t, t+T)$. The covariance of neuron i and j's spike counts is

$$\mathrm{Cov}(n_i^T, n_j^T) = \langle n_i^T n_j^T \rangle - \langle n_i^T \rangle \langle n_j^T \rangle, \tag{1}$$

where $\langle \cdot \rangle$ denotes an average over trials. The variance of neuron i's spike count is $\mathrm{Var}(n_i^T) = \mathrm{Cov}(n_i^T, n_i^T)$. The correlation coefficient of spike counts is

$$\mathrm{Corr}(n_i^T, n_j^T) = \frac{\mathrm{Cov}(n_i^T, n_j^T)}{\sqrt{\mathrm{Var}(n_i^T)\mathrm{Var}(n_j^T)}} \tag{2}$$

Here, we estimate spike count correlations by computing $\mathrm{Cov}(n_i^T, n_j^T)$ via the renewal relation [**?**]:

$$\mathrm{Cov}(n_i^T, n_j^T) = \int_{-T}^{T} \mathbf{C}_{ij}(s)\,(T - |s|)\,ds - r_i r_j. \tag{3}$$

and similar for $\mathrm{Var}(n_i^T)$. We examine this estimate for the average spike count correlation (over pairs of neurons in the network) as a function of window size. For the internally generated covariability in the main paper, spike count correlations are low (S1 Fig 1A).

Here, we write the linear response theory with external input correlations, for completeness. Specifically the fluctuating external input to each neuron was the sum of a private term and a globally shared term, $g_L \sigma D \left(\sqrt{(1-c)}\xi_i(t) + \sqrt{c}\xi_c(t)\right)$ (here, $\xi_i(t)$ and $\xi_c(t)$ are Gaussian white noise of unit intensity and g_L, σ and D defined as in Methods). The covariance matrix of the external inputs was $\mathbf{C}^{\mathrm{ext}}$, with $\mathbf{C}^{\mathrm{ext}}_{ij} = c g_L \sigma D$ for $i \neq j$ and $\mathbf{C}^{\mathrm{ext}}_{ii} = 1$. With correlated external inputs, the full spike-train cross-covariance matrix is given (in the Fourier domain) by [**?**]

$$\mathbf{C}(\omega) = \Big(\mathbf{I} - \big(\mathbf{W} \cdot \mathbf{K}(\omega)\big)\Big)^{-1} \Big(\mathbf{C}^0(\omega) + \mathbf{A}(\omega)\mathbf{C}^{\mathrm{ext}}(\omega)\mathbf{A}^*(\omega)\Big) \Big(\mathbf{I} - \big(\mathbf{W} \cdot \mathbf{K}^*(\omega)\big)\Big)^{-1} \tag{4}$$

As in the main text, $\mathbf{W}$ is the weight matrix, $\mathbf{K}_{ij}(\omega) = \mathbf{A}_i(\omega)\mathbf{J}_{ij}(\omega)$ is the effective interaction matrix, $\mathbf{A}(\omega)$ is a diagonal matrix containing the linear response function of each neuron. In the main text, what we refer to as the "baseline correlation" here corresponds to $\mathbf{C}^0(\omega) + \mathbf{A}(\omega)\mathbf{C}^{\mathrm{ext}}(\omega)\mathbf{A}^*(\omega)$.

Expanding the spike-train covariances in powers of the interactions $\mathbf{K}$ and truncating at first order yields the approximation:

$$\begin{aligned}\mathbf{C}_{ij}(s) \approx{}& \left(\mathbf{A}_i * \mathbf{C}^{\mathrm{ext}} * \mathbf{A}_j^-\right)(s) + \left(\mathbf{W}_{ij}\mathbf{K}_{ij} * \mathbf{C}^0_{jj}\right)(s) + \left(\mathbf{C}^0_{ii} * \mathbf{W}_{ji}\mathbf{K}^-_{ji}\right)(s) \\ &+ \sum_k \Big(\mathbf{W}_{ik}\mathbf{K}_{ik} * \mathbf{C}^0_{kk} * \mathbf{W}_{jk}\mathbf{K}^-_{jk}\Big)(s)\end{aligned} \tag{5}$$

We compare the spike count correlations for the $N = 1000$ network of Fig 2 in the main text to a network with $N = 100$ and synaptic weights ($W^{\max}$) increased by a factor of 10 (S1 Fig 1B). As in Figs. 2 and 3, this network was generated with Erdös-Rényi adjacency matrix and all weights had the same initial value, $W^{\max} * .6$ In this smaller network, with stronger synaptic weights, spike count correlations were larger, with an average long-window correlation of .005, and .03 in monosynaptically connected pairs.

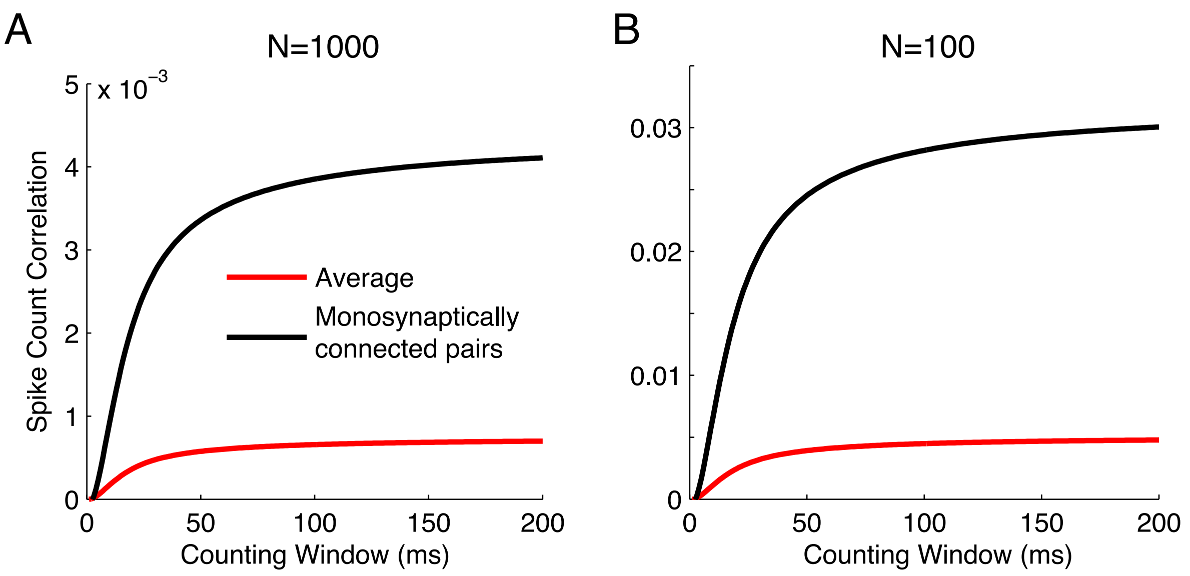

Figure 1. Magnitude of spike count correlations. Average spike count correlation as a function of the counting window size. (A) the network of Fig. 2. Spike count correlations are computed via the renewal relation, Eq. (3) using the first-order truncated approximation of the spike train cross-covariance function, Eq. (5). (B) A network of 100 neurons, with $W^{\max}$ increased by a factor of 10. Red, average spike count correlation across all pairs of neurons. Black, average spike count correlation in monosynaptically connected pairs.
