# Supplementary material for: Self-Organization of Microcircuits in Networks of Spiking Neurons with Plastic Synapses
Source: PLoS Comput Biol. 2015 Aug 20;11(8):e1004458. doi: 10.1371/journal.pcbi.1004458 (PMC4546203; doi:10.1371/journal.pcbi.1004458)
Supplement: S2 Text — (PDF) [file pcbi.1004458.s002.pdf]

Supplemental Material S2 Text: Strength of synaptic weights and stability of firing rates

Here, we varied the initial synaptic strength $p(0)$ and compared our theoretical predictions to empirical measurements from simulations for the mean synaptic strength $p(T)$, divergent $q^{\rm div}(T)$, convergent $q^{\rm con}(T)$, and chain $q^{\rm ch}(T)$ motifs, where $T = 1000$ min. For $p(0) \leq 5 \times 10^{-3}$ there was very good agreement between our reduced theory and spiking network simulations. When $p(0) \geq 7 \times 10^{-3}$ we observed a clear quantitative disagreement between theory and simulations. Increasing $p(0)$ or $p(T)$ above 8×10^{-3} caused an instability in the recurrent network, with runaway excitation destabilizing the equilibrium state, preventing any perturbative theory. The stability boundary for the stationary firing rates is computed as in [1], by computing the eigenvalues of the network firing rate from a Fokker-Planck theory [2]. Throughout the manuscript we always used $p(0) \leq 5 \times 10^{-3}$ to ensure agreement between simulations and theory.

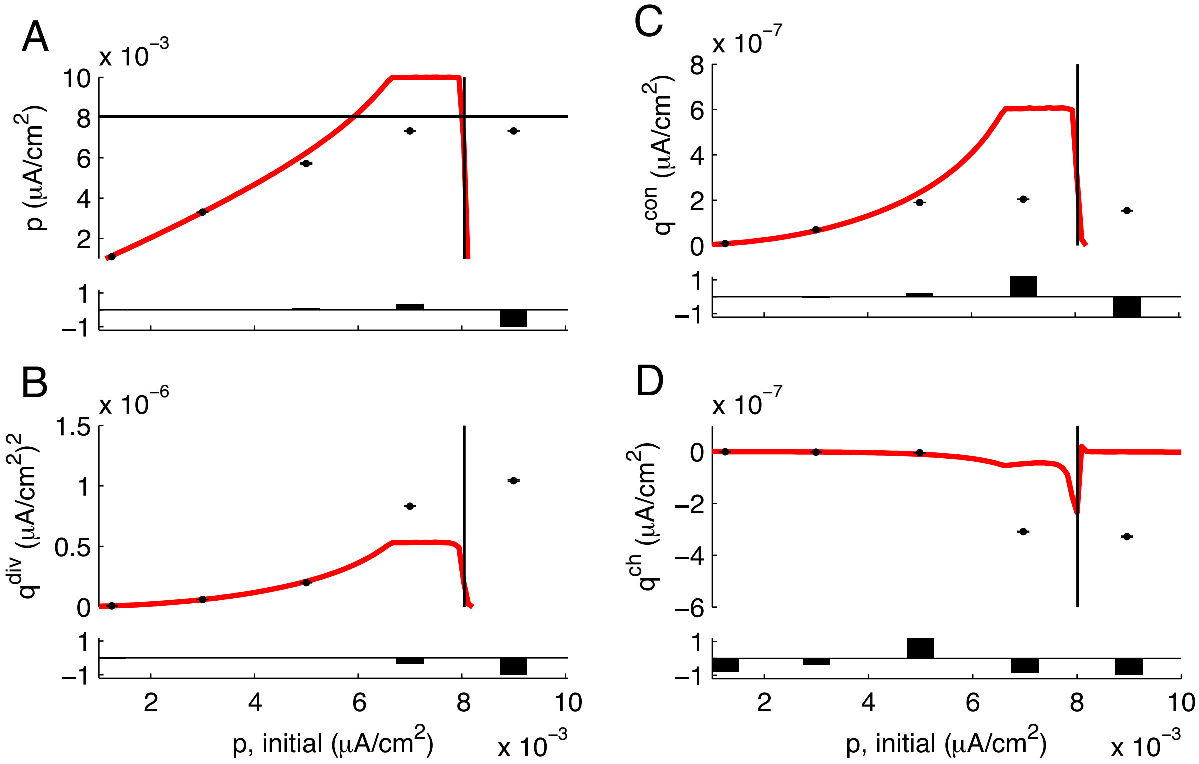

Figure 1. STDP in networks with larger synaptic weights. All panels: Red, motif theory of Eqs. (42)-(50) in main text. Black dots: result of spiking simulations. Error bars (smaller than marker size) are SEM. Black bars in lower part of each panel are the relative error of the theory to the simulations, (theory-sims)/(sims). (A) Final mean synaptic weight as a function of initial synaptic weight. Vertical and horizontal black lines mark the stability boundary for the stationary firing rates. For p beyond that boundary, we cannot use linear response theory for spike-train covariability (since there is no fixed point to linearize around) and so the theory will not match simulations. (B) Final strength of divergent motifs versus initial mean synaptic weight. (C) Final strength of convergent motifs versus initial mean synaptic weight. (D) Final strength of chain motifs versus initial mean synaptic weight.

References

1. Ledoux E, Brunel N (2011) Dynamics of networks of excitatory and inhibitory neurons in response to time-dependent inputs. Frontiers in Computational Neuroscience 5.

2. Richardson M (2007) Firing-rate response of linear and nonlinear integrate-and-fire neurons to modulated current-based and conductance-based synaptic drive. Phys Rev E 76: 021919.
