# Supplementary material for: Self-Organization of Microcircuits in Networks of Spiking Neurons with Plastic Synapses
Source: PLoS Comput Biol. 2015 Aug 20;11(8):e1004458. doi: 10.1371/journal.pcbi.1004458 (PMC4546203; doi:10.1371/journal.pcbi.1004458)
Supplement: S3 Text — (PDF) [file pcbi.1004458.s003.pdf]

Supplemental Material S3 Text: Motif plasticity in homogenous networks

If all neurons have the same weighted in- and out-degrees, then the motif dynamics simplify considerably. In such a homogenous network, divergent motifs obey:

$$\begin{aligned}\frac{dq^{\text{div}}}{dt} &= \frac{2}{N^3}\sum_{i,j,k}\mathbf{W}_{ik}\frac{d\mathbf{W}_{jk}}{dt} - 2p\frac{dp}{dt} \\ &= \frac{2}{N^3}\sum_{k}\left(\sum_{i}\mathbf{W}_{ik}\sum_{j}\frac{d\mathbf{W}_{jk}}{dt}\right) - 2p\frac{dp}{dt} \\ &= \frac{2}{N^3}Np\sum_{j,k}\frac{d\mathbf{W}_{jk}}{dt} - 2p\frac{dp}{dt} \\ &= 2p\frac{dp}{dt} - 2p\frac{dp}{dt} = 0\end{aligned} \quad (1)$$

Convergent and chain motifs are also neutrally stable. So, it is inhomogeneities in the network structure that give rise to drift of the motif structure.
