# Supplementary material for: Self-Organization of Microcircuits in Networks of Spiking Neurons with Plastic Synapses
Source: PLoS Comput Biol. 2015 Aug 20;11(8):e1004458. doi: 10.1371/journal.pcbi.1004458 (PMC4546203; doi:10.1371/journal.pcbi.1004458)
Supplement: S4 Text — (PDF) [file pcbi.1004458.s004.pdf]

Supplemental Material S4 Text: Motif plasticity with weight-dependent (multiplicative) STDP

The multiplicative STDP rule [1, 2] is:

$$\epsilon L(s) = \begin{cases} (\epsilon W^{\max} - \mathbf{W}_{ij}) f_+ e^{-\frac{|s|}{\tau_+}}, & \text{if } s \geq 0 \\ (\mathbf{W}_{ij})\,(-f_-)\, e^{-\frac{|s|}{\tau_-}}, & \text{if } s \leq 0, \end{cases} . \tag{1}$$

Each weight has a stable fixed point:

$$\mathbf{W}^*_{ij} = \mathbf{W}^0_{ij} \frac{f_+\tau_+}{f_+\tau_+ + f_-\tau_-} W^{\max} \tag{2}$$

Now the mean weight and two-synapse motifs evolve according to:

$$\epsilon \frac{dp}{dt} = \frac{1}{N^2} \sum_{i,j} \mathbf{W}^0_{ij} \int_{-\infty}^{\infty} \epsilon L(s) \left(r_i r_j + \delta_{ij} \mathbf{C}^0_{ij}(s) \right) ds \tag{3}$$

$$\epsilon^2 \frac{dq^{\mathrm{div}}}{dt} = \frac{2}{N^3} \sum_{i,j,k} \mathbf{W}_{ik} \mathbf{W}^0_{jk} \int_{-\infty}^{\infty} \epsilon L(s) \left(r_j r_k + \delta_{jk} \mathbf{C}^0_{jk}(s) \right) ds - 2\epsilon^2 p \frac{dp}{dt} \tag{4}$$

$$\epsilon^2 \frac{dq^{\mathrm{con}}}{dt} = \frac{2}{N^3} \sum_{i,j,k} \mathbf{W}_{ik} \mathbf{W}^0_{ij} \int_{-\infty}^{\infty} \epsilon L(s) \left(r_i r_j + \delta_{ij} \mathbf{C}^0_{ij}(s) \right) ds - 2\epsilon^2 p \frac{dp}{dt} \tag{5}$$

$$\epsilon^2 \frac{dq^{\mathrm{div}}}{dt} = \frac{2}{N^3} \sum_{i,j,k} \mathbf{W}_{ij} \mathbf{W}^0_{jk} \int_{-\infty}^{\infty} \epsilon L(s) \left(r_j r_k + \delta_{jk} \mathbf{C}^0_{jk}(s) \right) ds - 2\epsilon^2 p \frac{dp}{dt} \tag{6}$$

where we only examine the contribution of firing rates to the plasticity, assuming that the pre- and post-synaptic neurons' spike trains are uncorrelated. This corresponds to the observation that with multiplicative STDP, the weight-dependence of $L(s)$ dominates the dynamics of the weights. Inserting Eq. (1) and the motif definitions and assuming homogenous firing rates yields:

$$\frac{1}{\epsilon} \frac{dp}{dt} = r^2 \left(p_0 W^{\max} f_+\tau_+ - p\,(f_+\tau_+ + f_-\tau_-) \right) \tag{7}$$

$$\frac{1}{\epsilon} \frac{dq^{\mathrm{div}}}{dt} = 2r^2 \left(f_+\tau_+ W^{\max} q^{\mathrm{div}}_{\mathrm{X}} - (f_+\tau_+ + f_-\tau_-)\, q^{\mathrm{div}} \right) \tag{8}$$

$$\frac{1}{\epsilon} \frac{dq^{\mathrm{con}}}{dt} = 2r^2 \left(f_+\tau_+ W^{\max} q^{\mathrm{con}}_{\mathrm{X}} - (f_+\tau_+ + f_-\tau_-)\, q^{\mathrm{con}} \right) \tag{9}$$

$$\frac{1}{\epsilon} \frac{dq^{\mathrm{ch}}}{dt} = r^2 \left(f_+\tau_+ W^{\max} \left(q^{\mathrm{ch,A}}_{\mathrm{X}} + q^{\mathrm{ch,B}}_{\mathrm{X}} \right) - 2\,(f_+\tau_+ + f_-\tau_-)\, q^{\mathrm{ch}} \right) \tag{10}$$

The mixed divergent motifs obey:

$$\frac{dq^{\mathrm{div}}_{\mathrm{X}}}{dt} = r^2 \left(f_+\tau_+ W^{\max} q^{\mathrm{div}}_0 - (f_+\tau_+ + f_-\tau_-)\, q^{\mathrm{div}}_{\mathrm{X}} \right) \tag{11}$$

and $q^{\mathrm{con}}_{\mathrm{X}}$, $q^{\mathrm{ch,A}}_{\mathrm{X}}$ and $q^{\mathrm{ch,B}}_{\mathrm{X}}$ obey exactly analogous equations. Defining $q^{\mathrm{ch}}_{\mathrm{X}} = q^{\mathrm{ch,A}}_{\mathrm{X}} + q^{\mathrm{ch,B}}_{\mathrm{X}}$ puts the dynamics of q^{ch} into the same form as those for q^{div} and q^{con}. Dropping the motif labels, since they obey the same dynamics, yields a three-dimensional system for (p, q, q_{X}) with steady state

$$\begin{pmatrix} p \\ q \\ q_{\mathrm{X}} \end{pmatrix}^* = \begin{pmatrix} \frac{f_+\tau_+}{f_+\tau_+ + f_-\tau_-} p_0 W^{\max} \\ \left(\frac{f_+\tau_+}{f_+\tau_+ + f_-\tau_-} W^{\max} \right)^2 q_0 \\ \frac{f_+\tau_+}{f_+\tau_+ + f_-\tau_-} W^{\max} q_0 \end{pmatrix} \tag{12}$$

and Jacobian:

$$\begin{pmatrix} -(f_+\tau_+ + f_-\tau_-) & 0 & 0 \\ 0 & -(f_+\tau_+ + f_-\tau_-) & f_+\tau_+W^{\max} \\ 0 & 0 & -(f_+\tau_+ + f_-\tau_-) \end{pmatrix} \tag{13}$$

The eigenvalue of the three-dimensional multiplicative STDP system is $-(f_+\tau_+ + f_-\tau_-)$ which is always negative, so the steady state is linearly stable. So, multiplicative STDP simply stabilizes whatever motif structure is embedded in the adjacency matrix.

References

1. Rubin J, Lee D, Sompolinsky H (2001) Equilibrium properties of temporally asymmetric hebbian plasticity. Physical Review Letters 86: 364–367.

2. Van Rossum MC, Bi GQ, Turrigiano GG (2000) Stable hebbian learning from spike timing-dependent plasticity. The Journal of Neuroscience 20: 8812–8821.
