# Supplementary material for: Self-Organization of Microcircuits in Networks of Spiking Neurons with Plastic Synapses
Source: PLoS Comput Biol. 2015 Aug 20;11(8):e1004458. doi: 10.1371/journal.pcbi.1004458 (PMC4546203; doi:10.1371/journal.pcbi.1004458)
Supplement: S1 Fig — (PDF) [file pcbi.1004458.s005.pdf]

Supplemental Material S1 Fig: Plasticity in networks with larger correlations

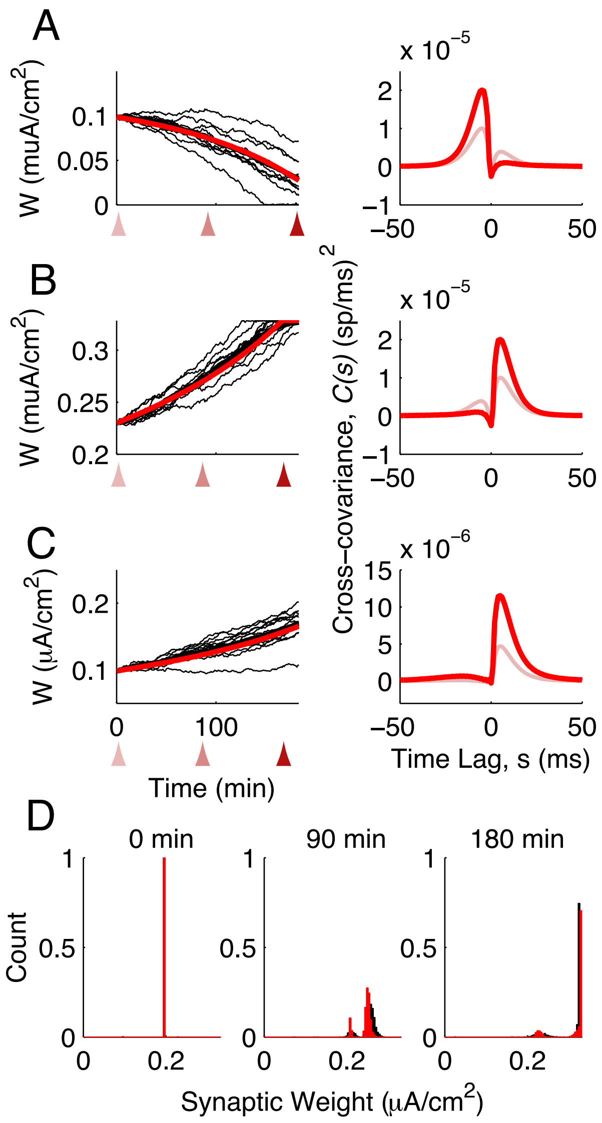

Figure 1. Plasticity in a network of 100 neurons. Here we examine synaptic plasticity in the $N = 100$ network of S1 Fig 1B. As an illustration, we embed the same three-neuron microcircuit as in Figs 2 and 3 of the main text into this network and examine the evolution of its synaptic weights and spike-train covariances . (A-C) Left, Synaptic weight versus time for each of the three synapses in the highlighted microcircuit of Figs. 2,3. Thin lines: simulation, individual trials of the same initial network. Thick black lines: simulation, trial-average. Thick red lines: theory. Right, spike train cross-covariances at the beginning and endpoints (linear response theory). (D) Histogram of synaptic weights at three time points. Red, theory. Black: simulation.
