# Supplementary material for: Self-Organization of Microcircuits in Networks of Spiking Neurons with Plastic Synapses
Source: PLoS Comput Biol. 2015 Aug 20;11(8):e1004458. doi: 10.1371/journal.pcbi.1004458 (PMC4546203; doi:10.1371/journal.pcbi.1004458)
Supplement: S2 Fig — (PDF) [file pcbi.1004458.s006.pdf]

Supplemental Material S2 Fig: Truncated vs full spike train cross-covariances

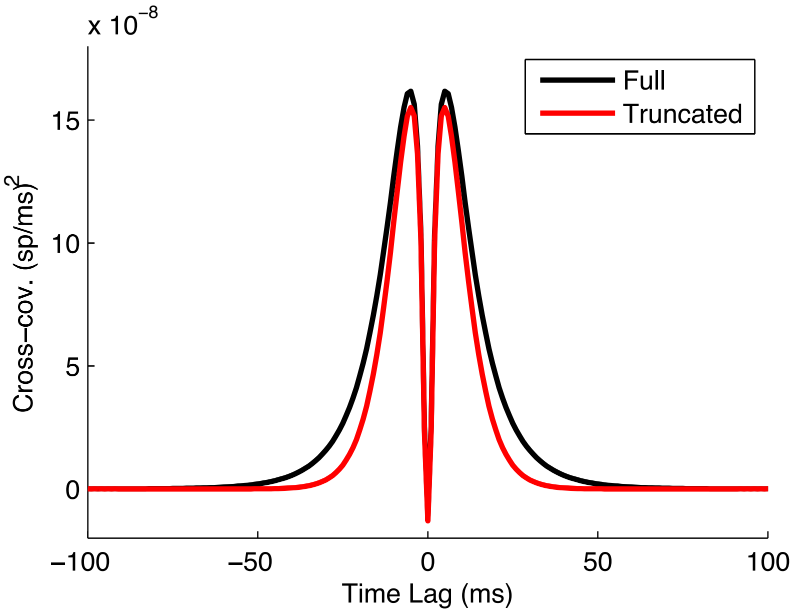

Figure 1. Truncated vs full spike train cross-covariance functions. Black, average spike train cross-covariance function of the network of Fig. 2. Red, truncated approximation (Eq. 7 of main text) of the average spike train cross-covariance function. When integrated against the balanced, depression dominated STDP rule, the full theory is 1.06 times as large as the truncated.
