# Supplementary material for: Self-Organization of Microcircuits in Networks of Spiking Neurons with Plastic Synapses
Source: PLoS Comput Biol. 2015 Aug 20;11(8):e1004458. doi: 10.1371/journal.pcbi.1004458 (PMC4546203; doi:10.1371/journal.pcbi.1004458)
Supplement: S3 Fig — (PDF) [file pcbi.1004458.s007.pdf]

# Supplemental Material S3 Fig: Balanced STDP in isolated pairs of neurons

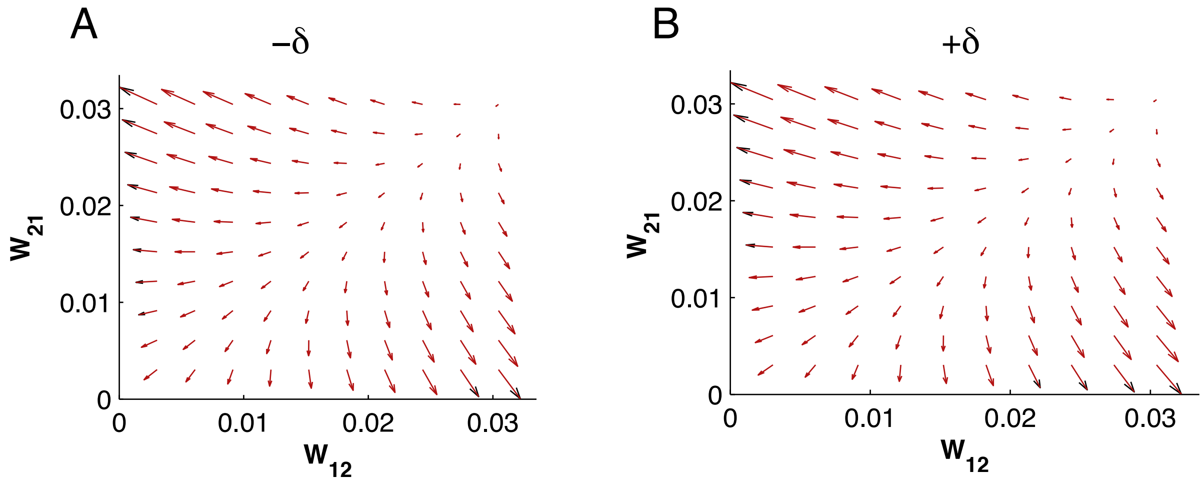


**Figure 1. Both types of balanced STDP lead to splitting of synaptic weights.** We take an isolated pair of neurons with the same intrinsic and synaptic parameters as in the full network. The neurons are reciprocally connected and we plot the (A) The depression-dominated balanced STDP rule. (B) The potentiation-dominated balanced STDP rule. Both are exactly the same as in the main paper. We show the dynamics of the weights in a pair of neurons isolated from the network. In both cases, the reciprocal loop is eliminated and initial conditions determine which synaptic weight is potentiated and which depressed.
