# Supplementary material for: Self-Organization of Microcircuits in Networks of Spiking Neurons with Plastic Synapses
Source: PLoS Comput Biol. 2015 Aug 20;11(8):e1004458. doi: 10.1371/journal.pcbi.1004458 (PMC4546203; doi:10.1371/journal.pcbi.1004458)
Supplement: S4 Fig — (PDF) [file pcbi.1004458.s008.pdf]

Supplemental Material S4 Fig: Rate-dependence of spike train covariability

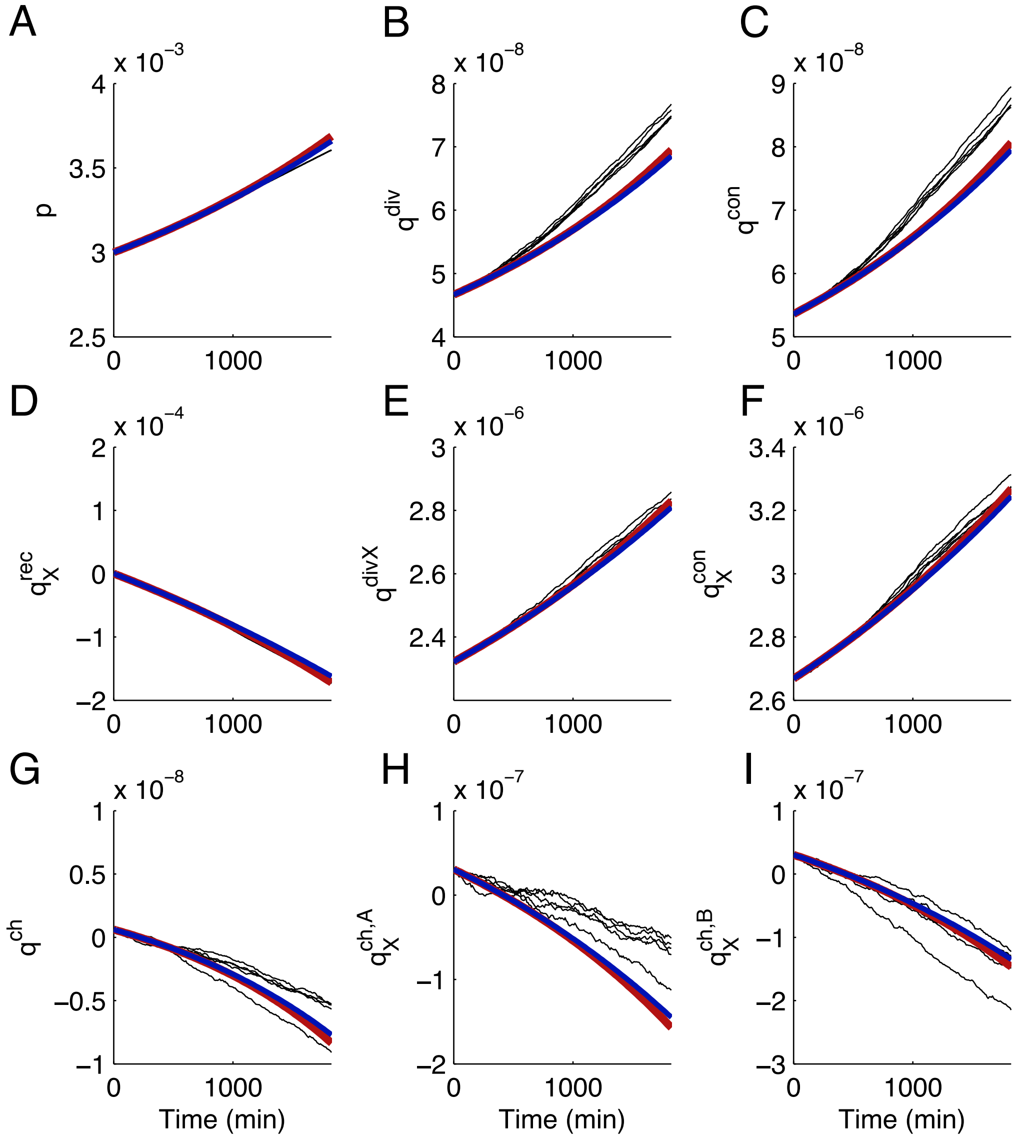

Figure 1. Motif plasticity does not depend on firing rates. In the main text, we examined the motif dynamics when the spike-train statistics are fixed. That is, for the motif theory we linearize the spike-train statistics around the initial state $r(t) = r(0)$, and similarly for the STDP-weighted spike train covariances S_F, S_B and S_C. Firing rates do, however, evolve with the mean synaptic weight. For $p = 0$, $r = 7.6$ sp/s while for $p = p_0 W^{\max}$, $r = 12.5$ sp/s. In order to check whether the approximation of using r, S_F, S_B and S_C calculated at initial conditions was accurate, we re-linearized the system at every time step of the slow evolution of synaptic weights so that the spiking statistics were not fixed. For the networks and parameters used here, this did not have a strong effect on the motif dynamics. We illustrate this by comparing the results of Fig. 7 to the motif dynamics with evolving spiking statistics. Red: motifs dynamics with fixed r, S_F, S_B, S_C - the spiking statistics are calculated by linearizing around the initial condition and held fixed. Blue: The spiking statistics (r, S_F, S_B, S_C) are re-linearized at every time step, so that the theory accounts for how firing rates and spike-train correlations depend on the net synaptic input neurons' receive.
